# A multi-epitope vaccine targeting a key virulence factor ClfA: An In-silico approach to combat *Staphylococcus aureus* infections

**DOI:** 10.1371/journal.pone.0334885

**Published:** 2025-10-24

**Authors:** Md. Nipatul Hasan Nirob, Ive Sultana, Tawsif al Arian, MD. Sazidul Islam, Md Moniruzzzaman, Safia Jerin Nosi, Sajal Kumar Halder, Aparna Shil, Mahbubul Kabir Himel

**Affiliations:** 1 Department of Pharmacy, Jahangirnagar University, Savar, Dhaka, Bangladesh,; 2 Department of Microbiology, Jahangirnagar University, Savar, Dhaka, Bangladesh; 3 Department of Botany, Jahangirnagar University, Savar, Dhaka, Bangladesh,; 4 Department of Biochemistry and Molecular Biology, Jahangirnagar University, Savar, Dhaka, Bangladesh; 5 Padma Bioresearch, Dhaka, Bangladesh; National Institute of Biologicals (NIB), Ministry of Health & Family Welfare, Government of India, INDIA

## Abstract

*Staphylococcus aureus,* a gram-positive opportunistic pathogen, presents a growing global threat due to the rise of multidrug-resistant (MDR) strains. To counter this, we designed a multi-epitope vaccine (MEV) targeting the ClfA virulence protein using an integrative in silico approach. Sixty-one conserved epitopes (19 CTL, 36 HTL, 6 LBL) were selected based on antigenicity, immunogenicity, non-toxicity, and lack of homology to human proteins. These epitopes demonstrated strong HLA-binding affinities and over 50% global population coverage, indicating broad immunological applicability. Molecular docking revealed the strongest binding between the MEV and TLR4, with a ΔG of –17.1 kcal/mol and an exceptionally low dissociation constant (2.6 × 10 ⁻ ¹² M). HADDOCK 2.4-supported docking scores corroborated these results. Molecular dynamics (MD) simulations and MM/GBSA analysis further assessed the structural behavior of the MEV in complex with TLR2, TLR3, and TLR4. While TLR2 and TLR3 complexes showed greater structural stability (RMSF ~0.2–0.5 nm), the TLR4 complex exhibited higher flexibility (RMSF ~2.5 nm) but yielded the most favorable binding free energy (ΔG = –174.41 kcal/mol), suggesting stronger overall interaction. The TLR2–vaccine complex formed ~370–400 hydrogen bonds on average, while the unbound vaccine maintained ~60–70 internal hydrogen bonds, confirming structural integrity. Radius of gyration (Rg) and solvent-accessible surface area (SASA) analyses revealed that TLR2 and TLR3 binding induced compact and stable structures, whereas the TLR4 complex was more solvent-exposed and flexible. Disulfide bond engineering (VAL32–THR37 and PHE45–ASN64) enhanced vaccine stability, further supported by favorable physicochemical parameters (MW 54.67 kDa, pI 7.78, instability index 19.78). The low eigenvalue (3.63 × 10 ⁻ ⁶) indicated high structural mobility, associated with efficient energy absorption. Codon optimization (GC content 53.33%, CAI 0.96) predicted high expression potential in *E. coli*, and *in silico* cloning was successfully performed using the pET-28a(+) vector. Immune simulation demonstrated robust humoral and cellular responses, including elevated levels of IgM, IgG1, IFN-γ, and increased B and T cell populations. Collectively, these findings suggest that the designed MEV is structurally stable, immunogenic, and capable of eliciting a potent immune response, with TLR4 emerging as a promising innate immune target. Further experimental validation and in vivo studies are essential to confirm its efficacy and safety as a vaccine candidate against *S. aureus* infections.

## 1. Introduction

Multi-drug-resistant (MDR) is amongst the most significant public health issues today and a worldwide hazard to human health [1]. MDR is interpreted as an organism’s reduced sensitivity to or resistance to an antibiotic drug that was previously susceptible. [2] Antibiotics were discovered about 80 years ago and within this period, drug-resistant microbes have appeared, which has threatened the survival of this era [3]. Resistance has already become a concern, even though newly invented antibiotics have been used [4]. However, the extensive use of antibiotics in human treatments contributed to the emergence of harmful bacteria resistant to multiple antibiotics [5]. Antimicrobial resistance is a major threat to the public’s health and requires immediate, important actions [1,4]. At the same time that MDR bacteria have appeared, the resources needed for finding and testing new antibiotics have decreased. In February 2017, the World Health Organization (WHO) announced a global list of bacteria and other microbes for which new drugs needed to be created. Several major threats from bacteria were highlighted, chief among them the group of ESKAPE pathogens for which immediate action on new treatment was required [6]. EKSAPE includes highly resistant, dangerous pathogen: *E. faecium, K. pneumoniae, S. aureus, A. baumannii, and P. aeruginosa, Enterobacter* spp [7]. Conventional antibiotics do not control such bacteria because they use several methods for causing disease, can resist the host environment and grow rapidly [8]. Because of genetic mutations and the sharing of mobile elements, some microorganisms can now resist commonly used antibacterial agents [6].

Opportunistic pathogen Staphylococcus aureus, known to be a Gram-positive bacterium, is an important member of this group. Such conditions boost the occurrence of infections, encourage their spread and play a major role in developing resistance to therapeutic medicines [9]. Although it is a normal flora of the human body that resides in the mucous membranes, vagina, throat, or injured skin surfaces, this organism can seriously invade the skin, soft tissues, respiratory system, bones, and joints. S. aureus can cause both types of diseases, suppurative and non-suppurative (by toxic substances) [10,11]. It can enter the body and infect all kinds of soft tissue, resulting in problems such as impetigo, furuncles, carbuncles, cellulitis and scalded skin syndrome. Systemic infections that S. aureus can cause include bacteraemia, infective endocarditis, pneumonia associated with ventilators, infections linked to intravenous catheters, lung diseases like pneumonia and empyema, meningitis, osteomyelitis, urinary tract infections, toxic shock syndrome and infections of the gastrointestinal system. Frequently, it is associated with ongoing breathing infections in individuals with cystic fibrosis [12,13]. Because it spreads through the blood and can infect several body organs, it causes serious health problems. Besides, S. aureus uses multiple strategies to escape the host immune system which, along with its antibiotic resistance, lets it live and cause prolonged infections [14].

Penicillin previously worked well to treat infections brought on by S. aureus. After penicillin became available in the 1940s, S. aureus developed penicillin resistance by taking up a plasmid-encoded β-lactamase enzyme [15]. To address this issue, methicillin, a novel, semi-synthetic, narrow-range, beta-lactamase-resistant antibiotic, was introduced in 1959 to battle penicillin-resistant bacteria. Methicillin-resistant strains, however, appeared swiftly [9]. Various genes are responsible for drug resistance, such as norA (fluoroquinolone resistance), tet (tetracycline resistance), lmrS (MDR), mepAR (multidrug and tigecycline resistance), and arlRS (fluoroquinolone resistance) [16]. On chromosomes, mecA, blaZ, erm(A), ant (6)-Ia, aadD, aph(3’)-III, and tet(M) were among the most prevalent antibiotic resistance genes detected. In plasmids, the most frequently found genes were blaZ, tet(K), spc, erm(A), and erm(C) [17].

Over 700,000 individuals worldwide die from antibiotic-resistant diseases each year, and by 2050, that number is anticipated to rise to 10,000,000 [18,19]. If no novel drugs are developed or found, there won’t be any potent medicines that are expected to be accessible by 2050. This emphasizes the requirement to seek different approaches to manage pathogens that are immune to antibiotics [9].

Due to the large number of antibiotic-resistant Staphylococcus aureus strains, coming up with a vaccine is currently the best strategy to fight this pathogen [20]. The urgent need for an effective *S. aureus* vaccine has grown globally because more types of bacteria are resistant to antibiotics [21]. Vaccines protect people from infections and help cut costs for healthcare services [22]. For many years, vaccination has been valuable in preventing infectious diseases and increasing overall immune system function. Because of this, health problems and losses have decreased, leaving people living longer. Progress in biotechnology has allowed vaccine development to deal with a larger number of diseases. Yet, traditional ways of creating vaccines are time-consuming, as they typically take between 5 and 15 years and are expensive too. Identifying the parts of a microbe that give rise to an immune response forms a basic part of designing vaccines. Thus, using in silico immunoinformatics tools makes vaccine development more practical and cost-effective than conventional methods [23]. Though conventional techniques have some drawbacks, using computational methods can now fix these problems [24]. Compared to traditional wet-lab methods, which may take months to years and require high cost for peptide synthesis, in vitro screening, and animal trials in the early phases, this computational strategy can be completed within weeks at a fraction of the cost. The increasing importance of immunoinformatic techniques makes it clear that advanced computing resources are more urgent than ever [25]. In silico approaches allow rapid screening, safety assessment, and structural validation before committing to expensive laboratory work, significantly reducing both development time and financial investment while minimizing resource expenditure and limiting unnecessary animal testing; these methods speed up the testing of new vaccines [26,27]. This makes computational vaccine design an efficient, cost-effective, and ethically preferable starting point before advancing to experimental validation. Vaccines made from epitopes cause fewer side effects and have the potential to treat more diseases. These vaccines trigger cellular and antibody defences by stimulating specific parts of microbes [23,28]. Current vaccine approaches are now based on picking out specific immunogenic regions instead of using whole, weakened germs [29]. The use of immunoinformatics allows for the prediction of CTL, HTL, and LBL epitopes, which is beneficial for vaccine design, assessing their effects, simulating the immune response, and checking their dependability [23]. To improve the weak immune response seen in single-epitope vaccines, experts developed the idea of designing multi-epitope vaccines. Multi-epitope vaccines are safer, offer a stronger immune response, cover more types of epitopes and are more easily taken up by antigen-presenting cells (APCs) [30]. Therefore, multi-epitope vaccines have better success rates than vaccines that contain a single epitope. For vaccines to be made, it is essential to look for suitable antigens, epitopes, peptide linkers and adjuvants in the computational pipeline [28]. As S. aureus is very immune to most antibiotics, developing new vaccines by computer methods is very important for handling related infections [31]. As a result, our key goal for this study is to develop a strong multi-epitope vaccine (MEV) against this harmful MDR bacteria. Using an immune protein from *S. aureus* and different software, we have introduced ideas for a vaccine that could greatly strengthen human immunity.

## 2. Methods and materials

**Fig 1:**
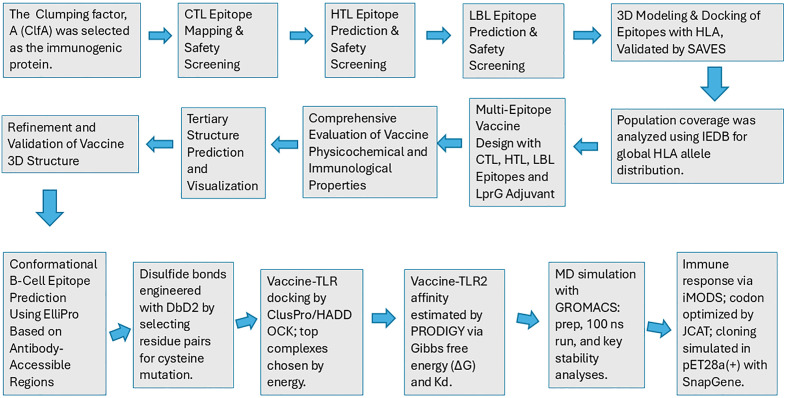
End-to-End in silico workflow employed in the multiepitope vaccine development.

An integrative in silico approach was employed to design, develop and evaluate the proposed multiepitope vaccine. It includes a systematic, multi-phase computational strategy in which diverse bioinformatics, immunoinformatics, and molecular modeling methods are sequentially and complementarily applied to design and assess a multi-epitope vaccine [32,33]. In this methodology, outputs from one analytical stage serve as inputs for subsequent steps, ensuring that predictions are refined and cross-validated at each stage [34].Many tools in our in-silico pipeline rely on robust machine learning algorithms to enhance predictive accuracy. Fig 1 represents the complete end-to-end computational pipeline used in this study, starting from sequence selection and data preprocessing, through prediction and filtering steps, and concluding with structural analysis and simulation.

### 2.1.1. Analysis of the antigen by immunological approach.

First, the clumping factor was chosen as the immunogenic protein in S. aureus, as it helps the bacteria stick to fibrinogen and other blood plasma proteins. The FASTA formatted amino acid sequence of this protein was attained from Uniprot (https://www.uniprot.org/). UniProt is a famous database and freely accessible to the public [35]. With a threshold value of 0.5, appropriate for bacterial organisms, the VaxiJen v2.0 server (https://www.ddg-pharmfac.net/vaxijen/VaxiJen/VaxiJen.html) was used to evaluate the antigenic potential of the sequences [36]. VaxiJen uses auto-cross covariance (ACC) transformation of amino acid physicochemical properties followed by partial least squares discriminant analysis (PLS-DA) for antigen prediction [36].

### 2.1.2. Epitopes for Cytotoxic T-lymphocytes (CTL) forecast and evaluation.

Epitopes consisting of 9 amino acids for cytotoxic T lymphocytes (CTLs) were found for ClfA using the NetCTL v1.2 web server (https://services.healthtech.dtu.dk/services/NetCTL-1.2/). All 12 MHC class I supertypes were used to make predictions after the FASTA-formatted sequences were uploaded. The standard configurations were carried over for C-terminal cleavage site prediction, TAP transport efficiency and epitope identification threshold. Scoring for rank was made by integrating the scores for MHC-I binding, proteasome cleavage and TAP uptake. It depends on artificial intelligence and weight matrix models to simulate the binding of peptides to MHC class I molecules, their transport by TAP and their digestion by the proteasome [37].

The CTL epitopes were chosen based on their capacity for induction of immune response, antigenicity, specificity toward allergens, homology, and toxicological properties. Several servers were employed for this purpose, including IFNepitope (http://crdd.osdd.net/raghava/ifnepitope/predict.php) for immunogenicity, [38] VaxiJen v2.0 server (https://www.ddg-pharmfac.net/vaxijen/VaxiJen/VaxiJen.html) for antigenicity, [36] AllerTOP v. 2.0 (https://www.ddg-pharmfac.net/AllerTOP) for allergen-specificity, [39] NCBI BLAST (Basic Local Alignment Search Tool), (https://blast.ncbi.nlm.nih.gov/Blast.cgi?PAGE=Proteins) for homology, [40] and ToxinPred (http://crdd.osdd.net/raghava/toxinpred/) for toxicity prediction [41] of CTL peptide/epitope. ToxinPred and AllerTOP v2.0 employ machine learning approaches such as Support Vector Machines (SVM) and QSAR-based classifiers, utilizing features like amino acid composition, physicochemical properties, and sequence-derived motifs to predict toxicity and allergenicity with high accuracy [42,43]. Analyzing proteins by using the ACC approach allowed us to generate special patterns that helped us detect important protein features. This method is sometimes used in QSAR research to predict the effects of epitope length on activity [44].

### 2.1.3. Epitope prognosis and assessment for helper T lymphocytes (HTL).

IEDB’s MHC-I/II binding predictors apply artificial neural networks (ANNs) and stabilized matrix method (SMM) models for epitope identification [45]. After screening ClfA sequences, the results were then analyzed through the IEDB MHC-II binding tool (http://tools.iedb.org/mhcii/) to look for potential helper T lymphocyte (HTL) epitopes [46]. Each HTL peptide of 15 amino acids was predicted using Consensus 2.22, concentrating on the HLA-DR region in humans. After ranking the peptides by their percentile scores, those with a score ≤2 was considered strong binders and chosen for additional investigation. Immunogenicity, antigenicity, allergenicity, homology, and toxicity were investigated in the same way as the CTL epitopes were analyzed. Significant HTL peptides were antigenic, not carriers of allergens, not toxic, homologous, and induced cytokine production. In addition, the cytokine release was investigated using the IL4pred (https://webs.iiitd.edu.in/raghava/il4pred/) [47] and IL10pred (https://webs.iiitd.edu.in/raghava/il10pred/) [48] servers. These servers operate using the SVM protocol, and the peak values of IL4pred and IL10pred are 0.2 and −0.3, respectively.

### 2.1.4. Epitopes for linear B-lymphocytes (LBL) prognosis and evaluation.

Computational approaches are usually applied to locate linear parts of antigens that are recognized by B-cell receptors. The prediction of these epitopes has become necessary for creating efficient subunit vaccines and activating the humoral immune system. Identifying B-cell-derived peptides is essential for the creation of vaccines that target specific parts of a protein. To find potential linear B-cell epitopes for vaccines, we used the ABCpred web server (http://crdd.osdd.net/raghava/abcpred/) [49]. The platform finds potential epitopes using artificial intelligence algorithms. A peptide containing 15 amino acids and a threshold of 0.51 was used. All predicted epitopes were tested for sequence comparisons, their potential to be antigens, allergens or toxic, following the same rules as CTL epitope assessment.

### 2.1.5. Analysis of modelling of the peptide with molecular interaction.

Using molecular docking, the chosen antigenic epitopes were tested against HLA alleles predicted from the selected genes to validate their overall affinity and possible usefulness in vaccines.

The 3-dimensional structures of all the epitopes of CD8 + T lymphocytes and helper T lymphocytes were produced by the PEPFOLD v3.0 server (https://bioserv.rpbs.univ-paris-diderot.fr/services/PEP-FOLD3/) [50]. In this server, a structural alphabet is used where each letter stands for a particular structure of a protein segment at a time. To predict what the peptide’s structure will be, the letters in the text are matched against a simple force field and a greedy method. With the PEP-FOLD server, up to 36 amino acids in a sequence can be analyzed to predict their 3D structures. The program also lets you define limits, such as the presence of disulfide bonds and how close different residues are allowed to be. Peptides are explored in the PEP-FOLD3 server with 100 separate simulations, each examining a unique segment of their possible shapes. All the models built from the simulations are available in an archive provided by the server. Moreover, users can review what clusters the models are associated with and which of the top five clusters has the best confirmation [51]. The validation of all peptide-epitopes is performed by using the SAVES v6.0 website (https://saves.mbi.ucla.edu/) [52]. This website utilizes the validated structure of the peptide with the assistance of programs like ERRAT, Verify3D, and PROCHECK. It compares the predicted structure of the model and that of a protein known from an experimental point of view.

The ERRAT program compares the predicted structure of a protein with actual experimental results at different overlapping parts to assess model quality. With the VERIFY 3D tool, the similarity between the produced 3D model and its sequence of amino acids was tested. Furthermore, the modeled structure was subjected to stereochemical tests using PROCHECK which is intended for validating results from X-ray crystallography or NMR spectroscopy.

The HLA alleles are taken from the RCSB Protein Data Bank (https://www.rcsb.org/) [53] and the ClusPro server (https://cluspro.org) is employed for protein-protein docking [54].

### 2.1.6. Demographic coverage determination.

If genetic diversity is to be accounted for, it would be better to choose several epitopes with diverse HLA binding capability, which will ensure a greater proportion of the population benefiting from multi-epitope-based peptide vaccinations. Population coverage assessment is difficult, as the wide variety of MHC alleles leads different HLA genotypes to occur much more frequently in some ethnic and cultural groups than others.

Hence, favoring the racial group through vaccination could be the result of not sufficiently regarding the national interests. Evaluation of the population coverage of the developed vaccine was conducted utilizing the population coverage platform of the IEDB server (http://tools.iedb.org/population/) [55]. This analysis will ascertain the extent to which each epitope is covered by the distribution of HLA-binding alleles in datasets from various global locations.

### 2.2. Multi-epitope-based vaccine formulation and assessment

#### 2.2.1. Outline of the vaccine’s construction.

To develop the multi-epitope vaccine, three types of epitopes—CTL, HTL and LBL—were combined using suitable linkers and an adjuvant. Peptide-based vaccines commonly depend on the use of adjuvants to make the body’s immune response stronger. LprG was chosen from mycobacterial lipoproteins in this study because of its established ability to enhance the immune response. LprG is essential for moving lipids to the mycomembrane of *Mycobacterium tuberculosis* and removing it causes a decrease in how virulent the bacteria are [56]. An EAAAK linker was attached to the adjuvant to give it further spatial separation and help stabilize its structure. Using AAY and GPGPG for the linkers, the researchers fused CTL and HTL epitopes in single cancer antigens. Thanks to these linkers, the multipart vaccine remains structurally flexible and shows the antigens as required.

#### 2.2.2. Both primary and secondary structural assessment.

For determining the physicochemical features of the prepared vaccine, the ProtParam server (at https://web.expasy.org/protparam/) was employed to check the components such as molecular weight, instability index, aliphatic index, theoretical isoelectric point (pI), grand average of hydropathicity (GRAVY) and half-life in vitro and in vivo [57]. The VaxiJen v2.0 server was used to test how antigenic the vaccine was, the Immunogenicity prediction tool from the IEDB was used to test immunogenicity and the AllergenFP v1.0 server was used to test allergens in the vaccine. For solubility prediction, SOLpro adopts a two-stage architecture, employing a Support Vector Machine (SVM) classifier followed by a Naïve Bayes model [58]. Solubility which has an impact on protein expression, was examined using the SOLpro tool (http://scratch.proteomics.ics.uci.edu/) [58].

#### 2.2.3. Modelling of tertiary configuration.

The Alphafold2 web server (https://colab.research.google.com/github/sokrypton/ColabFold/blob/main/AlphaFold2.ipynb) was used to predict the tertiary structure for a constructed vaccine [59]. AlphaFold2 combines deep residual neural networks and attention-based transformers within an end-to-end differentiable model to predict protein tertiary structures with high accuracy [60]. A 3D representation of this structure was generated using BIOVIA Discovery Studio 2017 [61].

#### 2.2.4. Confirmation and purification of the 3d configuration.

The raw 3D structure of the vaccine construct was improved by the GalaxyRefine server (http://galaxy.seoklab.org/), which relies on protocols from CASP10—that is, side-chain repositioning and relaxation of the complete structure through molecular dynamics [62] The process makes the model more accurate both on a local scale and in its overall layout. The quality of the refined MEV structure was evaluated through Ramachandran plot analysis using the RAMPAGE server [63]. In addition, the quality of the overall model was assessed with the ProSA server (https://prosa.services.came.sbg.ac.at/prosa.php), which provides a Z-score to measure how accurate the 3D structure is against known experimental proteins [64].

#### 2.2.5. Selection for configurational B lymphocyte cell regions.

The ElliPro tool from the IEDB database was applied using its default settings (a score of 0.5 or higher and a distance of 6 Å or less) to determine the conformational B-cell epitopes [65]. ElliPro uses geometrical algorithms based on 3D structural protrusion indices, combined with residue clustering, to predict conformational B-cell epitopes [66]. To predict discontinuous B-cell epitopes, ElliPro finds antibody-accessible regions by looking at the protein structure, computing residue protrusion values and grouping nearby areas that are more likely to be affected by an antibody.

#### 2.2.6. Bisulfide editing of vaccinal protein.

To strengthen the structure of the vaccine, the protein was analyzed for disulfide bond formation using DbD2 (http://cptweb.cpt.wayne.edu/DbD2/) [67]. DbD2 uses geometric filtering and energy scoring to identify optimal disulfide engineering sites [68]. Disulfide modulator helps find pairs of residues whose positions are suitable for disulfide engineering according to distance, angle constraints and energy characteristics. The energy for disulfide bond formation should be no greater than 2.2 kcal/mol and the χ3 angle needs to be at least −87° and no more than +97°. Those residues meeting the requirements were picked and changed into cysteines through the “Create/View Mutants” function to add disulfide bonds to the protein model.

#### 2.2.7. Analysis of the vaccine’s interaction with the TLRs protein.

To trigger a response, the vaccine acted as a connecting molecule while Toll-like receptors (TLR-2, TLR-3, TLR-4) served as the receiving receptor. The TLR structures were directly sourced from the RCSB PDB database. The interaction between the vaccine and TLR immune receptors was studied using the ClusPro v2.0 tool (https://cluspro.bu.edu/) [54]. ClusPro combines rigid-body docking with clustering of low-energy poses using scoring functions derived from van der Waals, electrostatics, and DARS potentials [69].Through its algorithm, the ClusPro web server calculated the energy (ΔE) involved in their binding process.


ΔE = 0.40E rep − 0.40E att + 600E elec + 1.00E DARS

The energy term E DARS, in the equation, symbolizes the paired configuration-based energy derived from the Decoys as Reference State (DARS) approach. In contrast, E rep and E attr denote the repulsive as well as attractive components, respectively of the van der Waals interaction energy. Computational docking is used for predicting or modeling the ternary structure of a complex molecule, beginning from the structures of the individual biomolecules when it is free and separate from the complex [70,71].

HADDOCK applies a data-driven docking protocol combining ambiguous interaction restraints (AIRs) with rigid-body energy minimization and molecular dynamics refinement to generate and rank protein–protein complexes [72]. HADDOCK 2.2 was used to find the most likely shapes of different protein complexes, utilizing its strong hierarchical algorithm [72]. By using the HADDOCK cluster and e-NMR GRID hardware, the server manages to calculate the docking effectively. In general, it takes about several minutes to prepare for a docking run, but the actual docking process, together with modeling and scoring, can take several hours [71].

The final docked models were selected based on HADDOCK scores and cluster size, which reflect both binding affinity and conformational stability. Models with the lowest HADDOCK score, indicating favorable intermolecular interactions, were prioritized. Additionally, cluster size, representing the number of similar conformations within a cluster, was considered to ensure reproducibility and reliability of the docking pose.

For this study, clusters with HADDOCK scores below −100.0 ± 5.0 (arbitrary example values based on typical HADDOCK results) and cluster sizes greater than 10 members were chosen as top candidates for further analysis and molecular dynamics simulations [73].The top structures obtained by HADDOCK with lower intermolecular energies were selected [72]. The top docked compound was selected for motion modeling based on its energy value and performance.

#### 2.2.8. Calculation of the Gibbs free energy (G) and dissociation constant.

The dissociation constant (Kd) of the vaccine-TLR2 complex was determined using the PRODIGY web page (https://bianca.science.uu.nl//prodigy/) [74]. PRODIGY is a website that predicts dissociation constants (Kd) from the analysis of the three-dimensional structure by calculating the Gibbs free energy (ΔG) of binding, which can be transformed into Kd according to the equation, ΔG = −RTlnKd, as R being the gas constant and T being the temperature in Kelvin. It calculates binding affinities from structural features such as interfacial contacts and non-interacting surfaces, employs a linear regression model trained on experimental protein–protein interaction datasets [75]. Therefore, using this web page, an estimated Kd value is obtained. The estimated Kd value provides information about the strength of the interaction between the vaccine and the TLR2 receptor. The PRODIGY algorithm was used to calculate ΔG, which is given by [76]:


$$\begin{array}{cc} \Delta \text{G}\;=\; & \;\text{0}.\text{09459}\times (\text{IC charged}/\text{charged}\;\times \;-\text{1})\;+\;\text{0}.\text{10007}\times (\text{IC charged}/\text{apolar}\;\times \;-\text{1})\;+\;\text{0}.\text{19577}\\ & \times (\text{IC polar}/\text{polar}\;\times \;\text{1})\;+\;\text{0}.\text{22671}\times (\text{IC polar}/\text{apolar}\;\times \;-\text{1})\;+\;\text{0}.\text{18681}\\ & \times (\%\text{NIS apolar}\;\times \;\text{1})\;+\;\text{0}.\text{3810}\times (\%\text{NIS charged}\;\times \;\text{1})\;–\text{ 15}.\text{9433}. \end{array}$$

In this scenario the positive values are assigned to IC charged/charged and IC/polar while negative values are given to IC charged/nonpolar and IC polar/nonpolar. %NIS apolar and %NIS charged also receive positive values.

#### 2.2.9. Molecular dynamics simulation.

Protein and protein-ligand complex microscopic stability is assessed using molecular dynamics, an advanced automated simulation method. Structure, function, fluctuation, interaction, and behavior is demonstrated to do this. The behavior of the four complexes were investigated using GROMACS version 2025.1 for MD calculations [77]. CHARMM General Force Field parameterized protein content. The SwissParam server implemented ligand topologies [78]. The structures were vacuum minimized 2500 times using steepest descent to address steric issues. The SPC water model solvated the structure. After adding Na+ and Cl- ions, the gmx genion instrument balanced the system. This was done to ensure system electrical neutrality. After minimization, MD simulations went into production, NVT, and NPT. Two phases balanced the systems. First, a 100 picosecond NVT equilibration was done to maintain particle number, volume, and temperature. The procedure aimed to raise system temperature to 300 kelvin. The second stage involved a precise 100 picosecond NPT equilibration to achieve temperature, pressure, and particle number homogeneity. It was essential to maintain system density and pressure. The protein group’s location was limited by bond limitations on all bonds during simulations. The system entropy reduced because NVT and NPT restricted water molecules around the protein, relaxing them. Parrinello-Rahman barostat method and v-rescale thermostat for molecular dynamics [79]. The thermostat and barostat were adjusted for 100 picoseconds. To constrain covalent bonding, the Linear Constraint Solver application was used. Chemical bond interactions were handled using the sophisticated (Particle-Mesh Ewald) or PME approach. Every system has a 100-ns production run after equilibrium.

**Root mean square deviation (RMSD).** RMSD is a metric that quantifies the distance between frames. It is determined for every profile frame. The root mean square deviation of frame x is calculated.


$${\text{RMSD}}_{\text{X}}=\sqrt{\frac{\text{1}}{\text{N}}\sum_{\text{I}=\text{1}}^{\text{N}}{\left({\text{r}}_{\text{i}}^{\prime }\left({\text{t}}_{\text{x}}\right)\right)-{\text{r}}_{\text{i}}\left({\text{t}}_{\text{ref}}\right)}^{\text{2}}}$$

The chosen set contains a total of N atoms. The reference time, t ref, is typically set to the first frame, t = 0. The position of the chosen atoms in frame x, after reference frame alignment, is denoted as r’. Frame x is associated with capture time t x. All simulation trajectory frames are handled with expertise. The MD trajectory parameters were analyzed using XmGrace.

**Root mean square fluctuation (RMSF).** RMSF values can be used to determine the local flexibility based on residue displacements during the MD simulation. The Root Mean Square Fluctuation (RMSF) is useful for characterizing local changes along the protein chain. The RMSF for residue i is:


$${\text{RMSF}}_{\text{I}}=\sqrt{\frac{\text{1}}{\text{T}}\sum_{\text{I}=\text{1}}^{\text{T}}{\left({\text{r}}_{\text{i}}^{\prime }(\text{t})\right)-{\text{r}}_{\text{i}}\left({\text{t}}_{\text{ref}}\right)}^{\text{2}}>}$$

RMSF is determined over trajectory time T. The reference time is t ref. The position of residue i is ri, and its atoms are r’ after superposition on the reference. The angle bracketed average square distance is calculated over residue atom selection. Protein regions with the highest simulated fluctuation are shown by the peaks on this map. N- and C-terminal protein tails vary more than other regions.

**Radius of gyration (Rg).** When mass weighting is taken into account, the radius of gyration (RG) is generally defined as the root mean square distance of an atomic group from their shared center of mass. The compactness of a molecular structure is indicated by the radius of gyration (Rg), which evaluates the proximity of the atoms to the centre of mass. This analysis is crucial for assessing the system’s overall stability and folding characteristics over time.

**Solvent accessible surface area (SASA).** The Solvent Accessible Surface Area (SASA) was calculated using the gmx sasa module of GROMACS 2025.1 over the course of a 200 ns molecular dynamics trajectory. The analysis was performed on the full complex, and the SASA values were recorded at regular intervals to monitor changes in surface exposure. Default probe radius (0.14 nm) and solvent parameters were applied. This analysis helped assess the folding stability and surface fluctuations of the protein–ligand complex during simulation.

**Hydrogen bonds.** Intramolecular hydrogen bonds were analyzed using the gmx hbond tool in GROMACS 2025.1. The analysis focused on hydrogen bonds formed within the protein throughout a 100 ns molecular dynamics simulation. Default criteria for hydrogen bond distance (≤ 0.35 nm) and angle (≥ 120°) were used. Hydrogen bond counts were computed for each frame of the trajectory to evaluate structural stability and integrity over time.

**MMGBSA binding free energy.** To estimate the binding free energy of the protein, Molecular Mechanics/Generalized Born Surface Area (MM/GBSA) calculations were performed using the gmx_MMPBSA tool (v1.6.3), which integrates GROMACS with AmberTools to enable end-state free energy analysis [80]. The approach decomposes the binding free energy (ΔG _bind_) into molecular mechanical energy (van der Waals and electrostatic interactions) and solvation energies (polar and nonpolar contributions), based on the equation:


$$\Delta {\text{G}}_{\text{bind}}=\;\Delta {\text{E}}_{\text{vdW}}+\;\Delta {\text{E}}_{\text{ele}}+\;\Delta {\text{G}}_{\text{polar}}+\;\Delta {\text{G}}_{\text{polar}}$$

In this study, the **Generalized Born (GB)** implicit solvent model was used to evaluate polar solvation energy, employing the **igb = 5** parameter corresponding to the GB-Neck2 model, while the nonpolar component was calculated from solvent-accessible surface area using the LCPO method. Entropy contributions were neglected, as is commonly done in comparative binding affinity assessment [81].

A total of **2001 snapshots** were extracted at equal intervals from the 200 ns production trajectory (MD_center.xtc) of the protein-ligand complex. The calculations were conducted on three separate systems: the complex, the receptor (protein alone), and the ligand. The final ΔG _bind_ was computed as:


$$\Delta {\text{G}}_{\text{bind}}=\;{\text{G}}_{\text{complex}}-({\text{G}}_{\text{receptor}}+\;{\text{G}}_{\text{ligand}})$$

**Principal component analysis (PCA).** Principal Component Analysis (PCA) was performed to assess the dominant motions in the protein-protein complex during the molecular dynamics simulation. PCA reduces the dimensionality of atomic fluctuations by diagonalizing the covariance matrix of positional deviations after least-squares fitting to a reference structure, yielding eigenvectors (principal components) and eigenvalues (their amplitudes) [82,83].

GROMACS 2025.1 was used for the trajectory preprocessing and PCA execution. The atomic positional covariance matrix was generated using the command gmx covar on the Cα atoms of the protein, producing eigenvectors and eigenvalues. Subsequently, the gmx anaeig tool was employed to analyze the first three principal components with respect to RMS fluctuations, eigenvector contributions, and 2D trajectory projection.

All PCA plots were generated using Xmgrace. The eigenvectors represent directions of motion in conformational space, while eigenvalues correspond to their relative amplitudes.

**Dynamic cross-correlation matrix (DCCM).** To explore residue-level correlated motions within the protein-protein complexes, dynamic cross-correlation matrix (DCCM) analysis was performed using the C-alpha atoms of the protein from the 200 ns molecular dynamics (MD) trajectories. The MD trajectories were processed with **MDAnalysis** in Python, where displacement vectors from mean positions were computed for each residue over time. The normalized correlation coefficients between residue pairs were calculated according to the formula:


$${\text{C}}_{\text{ij}}=\;\frac{\left({\Delta \text{r}}_{\text{i}}.{\Delta \text{r}}_{\text{j}}\right)}{\sqrt{{(\Delta \text{r}}_{\text{i}}^{\text{2}}).{(\Delta \text{r}}_{\text{j}}^{\text{2}})}}$$

Values of **C**_**ij**_ range from **+1** (fully correlated motion) to **−1** (fully anti-correlated motion), with 0 indicating no correlation. The resulting matrices were visualized using **Seaborn** heatmaps, where red indicates positive correlation and blue indicates anti-correlation. This approach provides insight into the allosteric communication and long-range interactions modulated by ligand binding, as established in previous studies [84,85].

#### 2.2.10. Investigations involving immune simulation.

To analyze the reaction of our antigen to the immune system, we simulated and studied the outcome with iMODS (https://imods.iqfr.csic.es/) [86]. It uses Position-Specific Scoring Matrix (PSSM) techniques along with Artificial Intelligence to model the immune response, offering useful information about how the vaccine candidate might affect the immune system.

#### 2.2.11. In silico replication and codon modification.

We utilized the Java Codon Adaptation Tool (JCAT) web platform (http://www.Jcat.de/) to modify the genetic codons of the vaccine construct [87]. To evade prokaryotic ribosomal anchoring, rho-dependent transcription termination, and restriction enzyme degradation sites, three choices were selected from the list available at the bottom of the website. During this process, we determined the guanine-cytosine (GC) content and codon adaptation index (CAI) number of the modified sequence [51]. Being used optimally for expression, a CAI (Codon Adaptation Index) value of 1.0 is considered ideal and scores above 0.8 are generally thought to be satisfactory too [88]. The CAI allows us to examine if the codon usage pattern of a virus is biased relative to that of its host. To ensure the vaccine construct worked and to simulate its cloning process, the DNA optimized sequence was inserted into the MCS of pET28a(+) vector, using SnapGene v4.3 software [89].

## 3. Result

### 3.1 Pre-steps of vaccine construction

#### 3.1.1 Analysis of protein sequences and assessment of antigenicity.

For the ClfA protein, a total of 68 epitopes were found after the screening. Among them, 19 epitopes for CTL, 36 for HTL, and 13 for LBL were ultimately chosen according to their various properties. The 5 allele HLA-B*58:01, HLA-B*15:01, HLA-B*40:01, HLA-A*30:01, and HLA-A*32:01, as well as the 10 MHC supertypes A1, A2, A3, A24, A26, B7, B27, B39, B58, and B62, are the source of all chosen CTL peptides. For HTL, the immunogenicity, antigenicity, toxicity, and homology of distinct peptides are evaluated. In addition to other criteria, LBL is also evaluated for immunogenicity value.

**Fig 2:**
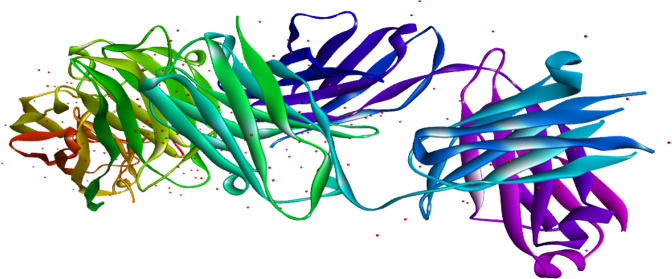
The 3D model of structural proteins of ClfA protein of *Staphylococcus aureus.*

We obtained the sequences of ClfA protein of *Staphylococcus aureus* from the NCBI Genbank (https://www.ncbi.nlm.nih.gov/genbank/) [90] (Fig 2) that has residues of 521 amino acids.

#### 3.1.2. Target glycoproteins and their antigenicity prediction.

The VaxiJen server found epitopes with antigenic property scores ranging from 0.4030 to 1.96 [36]. ProtParam was applied to analyze a variety of parameters [57]. The results showed that the protein had favorable antigenic properties and was composed of 521 amino acids with an atomic weight range of 54.67 kDa. The instability index less than 40 was found to be the stability threshold for all proteins linked to the Nucleocapsid glycoprotein, suggesting that all protein groups are stable. A thorough presentation of the physicochemical features was made (Table 1).

**Table 1:** Physical and chemical characteristics of the ClfA protein. Expasy Protparam tool and VaxiJen v2.0 server predicted the parameters.

Target Protein	Antigenicity (Threshold, 0.5)	Molecular Weight	Instability Index	Aliphatic Index	Theoretical Isoelectric Point	No. of Amino acids	Extinction Co-Efficient	Estimated half-life:	GRAVY
ClfA	**0.** 8626	z	19.78	73.42	7.77	521	51800	30 hours (mammalian reticulocytes, in vitro). >20 hours (yeast, in vivo). >10 hours (*Escherichia* *coli*, in vivo).	−0.861

#### 3.1.3. Epitope model prediction.

The PEP-FOLD method is a novel tool for predicting the shapes of peptides based on their sequences of amino acids. Every epitope targeting CTL, HTL, and LBL has been included in this simulation. For each epitope, the system generates 5 models, and the best model is then selected for closer investigation. In this comparison, the energy value of each model, quality standards, modelling structure, and experimental confirmations are considered.

For antigenicity, allergenicity, and toxicity predictions, confidence thresholds and validation metrics were employed to ensure reliability. For example, VaxiJen uses a default antigenicity threshold of 0.4 with reported accuracy up to 70–89% depending on the dataset [36]. AllerTOP v2.0 reports an accuracy of ~88% for allergen prediction using machine learning classifiers [43], while ToxinPred achieves an accuracy of ~85% in identifying toxic peptides [42]. To minimize false positives, a consensus-based approach was adopted by cross-validating predictions across multiple tools, thereby enhancing overall predictive confidence and robustness [91,92].

#### 3.1.4 Epitope forecasting of B-Cell and T-cell epitopes.

With strong binding affinities, the Epitopes to a scientifically validated shared allele are optimal choices for constructing MEV constructs. Another obstacle to predicting epitope binding to the HLA class I/II molecules is the restricted availability of prediction server tools. To identify the most likely epitopes with the highest binding affinity, we carefully curated each unique allele reported in the literature. Additionally, epitopes that bind to numerous alleles were thought to be the best candidates for their robust defence capacities.

#### 3.1.5 Estimation of CTL epitopes.

CTLs help protect the body by destroying cells that have become infected with bacteria and viruses [93]. Prediction of immunogenic CTL targets was carried out using NetCTL 1.2 server [37] based on binding to a wide range of MHC class I alleles [47]. Epitopes of about 9–12 amino acids after selection were selected for their high possible binding characteristics. A strong association between epitopes and MHC alleles was realized by predictive scores that were both high and accompanied by low percentile ranks. Nineteen of the 61 MHC class I alleles were chosen for study, and many had a high affinity for the ClfA protein epitopes (Table 2).

**Table 2:** Physicochemical characteristics of predicted CTL epitopes.

Residue Number	Epitope	Supertypes	Combined Score	Antigenicity	Allergenicity	Toxicity	Homology	Immunogenicity
314	YGYNSNIIW	B39	0.8201	0.4657	No	Non-toxin	Non-homologue	1.6955333
182	QQNTSIKVY	B62	1.4637	1.2191	No	Non-toxin	Non-homologue	1.6847608
43	SEDEANTSL	B39	1.9802	1.1827	No	Non-toxin	Non-homologue	1.6670375
54	RRKKENKDK	B27	1.3551	1.9600	No	Non-toxin	Non-homologue	1.6607211
187	VTNSVNITF	B58	1.6451	0.9582	No	Non-toxin	Non-homologue	1.6496116
253	QAGYVKLNY	B58	0.9337	0.8647	No	Non-toxin	Non-homologue	1.6395218
387	NSAVKGDTF	B58	0.823	1.1751	No	Non-toxin	Non-homologue	1.6221428
120	KPNTDSNAL	B7	1.6857	1.1168	No	Non-toxin	Non-homologue	1.5685302
59	DLALRSTLY	A26	1.3636	0.7717	No	Non-toxin	Non-homologue	1.5682245
290	SPQNSTNAE	B7	1.0739	1.6299	No	Non-toxin	Non-homologue	1.4090577
324	LYGYNSNII	A24	1.7327	0.6494	No	Non-Toxin	Non-homologue	1.4048082
110	GIGSTTANK	A3	1.2372	1.7079	No	Non-Toxin	Non-homologue	1.395761
67	TLYGYNSNI	A2	1.1942	0.7421	No	Non-Toxin	Non-homologue	1.2006163
45	STTANKTVL	B39	0.7719	1.1075	No	Non-Toxin	Non-homologue	1.1443479
299	GIDSGTTVY	A3	0.8249	0.8239	No	Non-Toxin	Non-homologue	0.98905271
154	NQTSNETTF	B62	0.9185	1.1202	No	Non-Toxin	Non-homologue	0.9495904
22	RMRAFSLAA	B62	1.1108	0.4531	No	Non-Toxin	Non-homologue	0.53026873
97	LIWGLLASI	A2	1.0844	0.8661	No	Non-Toxin	Non-homologue	0.47438779
65	STDASNKDV	A1	1.3979	1.6872	No	Non-Toxin	Non-homologue	0.2715526

#### 3.1.6 HTL epitope prediction.

Helper T lymphocytes (HTLs) are important for running both kinds of immune responses. These cells stand out because they have unique receptor-based HTL epitopes. Predictions on HTL epitopes were made for ClfA by using the NetMHC II pan 3.2 server [94]. The Table 3 data reveal that epitopes with higher percentile ranks are weaker immunologically. Positive results on the IFN-gamma prediction server indicated that certain epitopes are likely to prompt the release of interferon-gamma (Table 3) [95]. The vaccine process involved narrowly choosing eleven HTL epitopes to include in the construction.

**Table 3:** Physicochemical characteristics of predicted HTL epitopes.

Epitope	Antigenicity	Allergenicity	Toxicity	Homology	Immunogenicity
DDVKATLTMPAYIDP	0.8930	No	Non-toxin	Non-homologue	1.5544274
PHQAGYVKLNYGFSV	0.8043	No	Non-toxin	Non-homologue	1.5543947
YIVVVNGHIDPNSKG	0.7883	No	Non-toxin	Non-homologue	1.5544507
KFYNLSIKGTIDQID	0.7632	No	Non-toxin	Non-homologue	1.5544993
LIGFGLLSSKEADAS	0.7528	No	Non-toxin	Non-homologue	1.5544053
VNITFPNPNQYKVEF	0.7339	No	Non-toxin	Non-homologue	1.5544389
LNGVTSTAKVPPIMA	0.7295	No	Non-toxin	Non-homologue	1.5543947
MNMKKKEKHAIRKKS	0.6477	No	Non-toxin	Non-homologue	1.5545679
SKGDLALRSTLYGYN	0.5331	No	Non-toxin	Non-homologue	1.5544993
VNPENFEDVTNSVNI	0.4796	No	Non-toxin	Non-homologue	1.5544161
TTPYIVVVNGHIDPN	0.6779	No	Non-toxin	Non-homologue	1.5544274
EDEANTSLIWGLLAS	0.4838	No	Non-toxin	Non-homologue	0.44821515
NFEDVTNSVNITFPN	0.4340	No	Non-toxin	Non-homologue	1.5545679
ETPVTGEATTTTTNQ	1.4065	No	Non-toxin	Non-homologue	0.25856593
QYKVEFNTPDDQITT	1.2639	No	Non-toxin	Non-homologue	1.5544748
QAGYVKLNYGFSVPN	0.7672	No	Non-toxin	Non-homologue	1.5544389
KYGKFYNLSIKGTID	0.6722	No	Non-toxin	Non-homologue	1.5543654
PENVKKTGNVTLATG	0.6440	No	Non-toxin	Non-homologue	1.5543947
SEDEANTSLIWGLLA	0.5931	No	Non-toxin	Non-homologue	1.5543947
GTTVYPHQAGYVKLN	0.4126	No	Non-toxin	Non-homologue	1.5544274
VDYEKYGKFYNLSIK	0.5720	No	Non-toxin	Non-homologue	1.5544389
APRMRAFSLAAVAAD	0.5168	No	Non-toxin	Non-homologue	1.2679201
NTSIKVYKVDNAADL	0.5138	No	Non-toxin	Non-homologue	1.5543564
AGYVKLNYGFSVPNS	0.4030	No	Non-toxin	Non-homologue	0.39180078
YGYNSNIIWRSMSWD	0.4203	No	Non-toxin	Non-homologue	1.5544274
NALIDQQNTSIKVYK	0.4884	No	Non-toxin	Non-homologue	1.5543947
ALIDQQNTSIKVYKV	0.5304	No	Non-toxin	Non-homologue	0.10339732
GNVIYTFTDYVNTKD	0.4271	No	Non-toxin	Non-homologue	2.0964387
NVIYTFTDYVNTKDD	0.4838	No	Non-toxin	Non-homologue	1.5543654
VIYTFTDYVNTKDDV	0.4273	No	Non-toxin	Non-homologue	1.5543947
VPKELNLNGVTSTAK	0.9003	No	Non-toxin	Non-homologue	1.5544389
PKELNLNGVTSTAKV	1.0725	No	Non-toxin	Non-homologue	1.5544161
QQNTSIKVYKVDNAA	0.8101	No	Non-toxin	Non-homologue	1.5544627
QNTSIKVYKVDNAAD	0.7014	No	Non-toxin	Non-homologue	1.5543846
SGTTVYPHQAGYVKL	0.4313	No	Non-toxin	Non-homologue	1.5543846
DSGTTVYPHQAGYVK	0.4046	No	Non-toxin	Non-homologue	2.207793

#### 3.1.7 Preservation analysis, populace scope, and autoimmunity identification.

**Fig 3:**
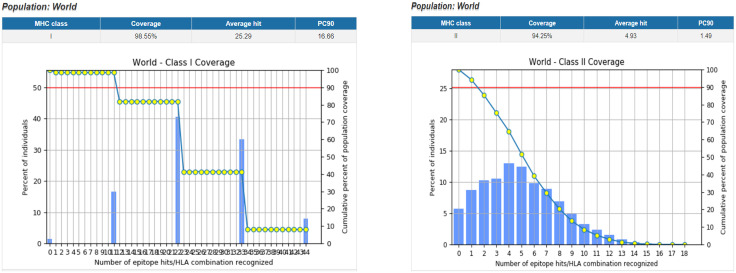
World population coverage of MHC class-I and MHC class-II.

All selected T cell and LBL epitopes (S1 Table) within relevant auxiliary proteins were 100% preserved, according to the preservation assessment. Furthermore, the examination of population coverage revealed that more than 50% of the world’s population (Fig 3) was represented by T-cell epitopes [95,96].

With a population coverage of over 50%, none of the chosen epitopes showed any homology with human proteins. Population coverage is a crucial factor in the creation of an MEV since the distribution of HLA alleles varies among different ethnic groups and geographical constituencies of the world. The current investigation calculated the total population coverage of the HTL and CTL epitopes that had been shortlisted, along with the accompanying HLA alleles. Briefly, our analysis proved that the chosen epitopes would be the ideal candidates for the creation of an MEV construct.

### 3.2. Vaccine construction and post-constructional studies

#### 3.2.1. Designing of a multiple-epitope vaccine.

**Fig 4:**
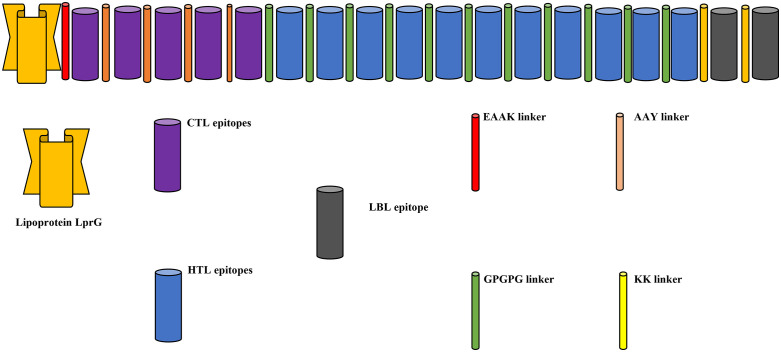
Schematic diagram of final vaccine construct.

For a vaccination program to be effective, helper T lymphocytes (HTLs) and cytotoxic T lymphocytes (CTLs) must both elicit immunological responses. Therefore, the vaccine formulation must have suitable, coupled CTL and HTL epitopes. The investigation used CTL and HTL screening methods to find MEVs. candidates with immune-stimulating qualities, persistent antigenicity, and lack of allergic reaction. The conjugation of CTL epitopes with linker molecules is reported in this paper and the GPGPG linker-mediated coupling of HTL epitopes. Furthermore, the connection between B-cell Linker KK was utilized to achieve epitopes using the adjuvant Lipoprotein LprG (Fig 4).

A multi-epitope immunization was developed, including 521 residues of amino acids, and then tested for physicochemical, allergenic, and antigenic qualities.

#### 3.2.2 mRNA structure prediction.

**Fig 5:**
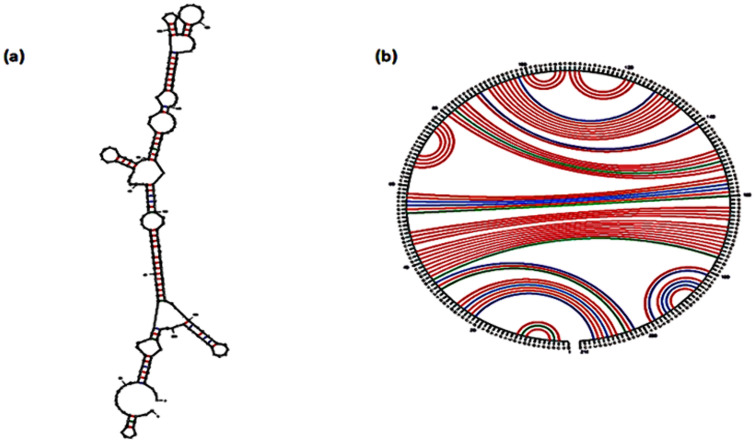
(a) Prediction of the RNA secondary structure of the multi-epitope vaccine construct gene by the Mfold server. (b) Circle graph structure, which displays the base pairs of the structure.

Predicting the secondary structure of mRNA (Fig 5) is a significant factor that can help with the calculation and interpretation of mRNA’s biogenesis, elongation, and initiation. We acquired statistical information on the free energy (Table 4) associated with the whole mRNA structure by utilizing the Mfold online tool. According to the score, the protein’s interpretation is both strong and effective [97].

**Table 4:** Thermodynamics of the manufactured vaccine’s folding.

Structural element	δG	Information
External loop	−0.2	498 ss bases & 1 closing helices.
Stack	−0.3	External closing pair is A ^279^-V ^288^
Stack	−1.20	External closing pair is A ^280^-U ^287^
**Helix**	−2.40	3 base pairs.
Hairpin loop	4.12	Closing pair is V ^281^-A ^286^

Table 4 provides the important thermodynamic properties of the RNA secondary structure of the vaccine construct. Both stacks and helices are shown to have lower δG values which reveals they are stable regions, while the loop in a hairpin has a positive δG, meaning it may not be as stable. Strong and efficient folding of the RNA transcript is possible because of these thermodynamic parameters, making it more stable and a good candidate for the vaccine.

#### 3.2.3 HLA allele-epitope interaction study.

After assessing the interaction among each chosen CTL and HTL epitope with distinct HLA alleles, sixteen docked complexes were found; five were from CTL and the remaining were from HTL (Tables 5 and 6). After being determined to be binding epitopes with the relevant HLA allele, epitopes exhibiting a sufficient global binding affinity threshold were selected for the absolute vaccine design. Additionally, two LBL epitopes were chosen for the construction of the MEV (S2 Table).

**Table 5:** Final Selected CTL Epitopes for MEV Design Based on Docking Analysis.

Serial No.	Protein	CTL	C-score	Antigenicity	IFN-gamma	Epitope Conservancy Hit (%)	Interacting MHC-I alleles
1	ClfA	YGYNSNIIW	0. 8201	0. 4657	0.0915378	100	HLA-A*30:02,HLA-A*32:01,HLA-B*57:01,HLA-B*35:01,HLA-A*30:01,HLA-B*08:01,HLA-A*30:02,HLA-B*53:01,HLA-A*31:01,HLA-B*51:01,HLA-B*08:01,HLA-B*53:01,HLA-A*03:01,HLA-A*01:01,HLA-B*58:01,HLA-A*68:02,HLA-B*51:01,HLA-A*30:01,HLA-A*23:01,HLA-B*44:02,HLA-A*02:06,HLA-B*57:01,HLA-B*07:02,HLA-B*58:01,HLA-A*01:01,HLA-A*03:01,HLA-A*24:02,HLA-A*68:02,HLA-B*40:01,HLA-A*11:01,HLA-B*40:01,HLA-A*26:01
2	ClfA	QQNTSIKVY	1.4637	1.2191	0.920684	100	HLA-A*02:01,HLA-A*26:01,HLA-A*30:01,HLA-B*08:01,HLA-A*30:02,HLA-B*44:02,HLA-B*15:01,HLA-A*24:02,HLA-A*02:03,HLA-A*11:01,HLA-A*02:01,HLA-A*03:01,HLA-A*02:03,HLA-A*33:01
3	ClfA	SEDEANTSL	1.9802	1.1827	0.256406	100	HLA-A*68:01,HLA-A*68:02,HLA-A*33:01,HLA-A*68:01,HLA-A*31:01,HLA-A*32:01,HLA-B*44:03,HLA-B*44:03
4	ClfA	RRKKENKDK	1.3351	1.9600	0.329762	100	HLA-B*53:01,HLA-B*51:01,HLA-B*58:01,HLA-A*68:02,HLA-A*26:01,HLA-A*01:01,HLA-B*57:01,HLA-B*40:01,HLA-B*44:02,HLA-B*15:01,HLA-A*24:02,HLA-A*11:01,HLA-A*02:01,HLA-A*03:01,HLA-A*02:03,HLA-A*33:01,HLA-A*02:06,HLA-A*23:01,HLA-A*68:01,HLA-A*31:01
5	ClfA	VTNSVNITF	1.6451	0.9582	0.875612	100	HLA-A*30:02,HLA-B*53:01,HLA-B*15:01,HLA-A*01:01,HLA-B*51:01,HLA-A*30:01,HLA-A*23:01,HLA-B*44:02,HLA-A*02:06,HLA-B*07:02,HLA-A*03:01,HLA-B*40:01,HLA-A*31:01,HLA-A*02:01,HLA-A*26:01,HLA-B*08:01,HLA-A*02:03,HLA-A*68:01

**Table 6:** Final epitopes (HTL) chosen following docking analysis with corresponding HLA alleles for MEV design.

SI No.	Protein	HTL	Antigenicity	IL-4	IL-10	IFN-gamma	Epitope Conservancy hit	Interacting MHC-II alleles
1	ClfA	DDVKATLTMPAYIDP	0.8930	Inducer	Non-inducer	0.110285	100	HLA-DRB1*07:01,HLA-DRB1*11:01,HLA-DRB1*13:02,HLA-DRB1*09:01,HLA-DRB1*02:05
2	ClfA	PHQAGYVKLNYGFSV	0.8043	Non-inducer	Non-inducer	0.038252	100	HLA-DRB1*12:01,HLA-DRB5*01:01,HLA-DRB1*15:01
3	ClfA	YIVVVNGHIDPNSKG	0.7883	Inducer	Inducer	0.441461	100	HLA-DRB1*02:02,HLA-DRB3*07:02
4	ClfA	KFYNLSIKGTIDQID	0.7632	Inducer	Non-inducer	0.371242	100	HLA-DRB1*01:01,HLA-DQA1*05:01,HLA-DQB1*03:01,HLA-DRB1*07:01,HLA-DRB1*02:01
5	ClfA	LIGFGLLSSKEADAS	0.7528	Non-inducer	Inducer	0.845046	100	HLA-DRB1*12:01,HLA-DRB5*01:01,HLA-DRB1*15:01,HLA-DRB1*11:01
6	ClfA	VNITFPNPNQYKVEF	0.7339	Non-inducer	Non-inducer	0.034832	100	HLA-DRB1*13:02,HLA-DRB5*01:01,HLA-DRB1*01:01,HLA-DRB1*08:02,HLA-DRB1*12:01,HLA-DRB1*09:01
7	ClfA	LNGVTSTAKVPPIMA	0.7295	Inducer	Inducer	0.322304	100	HLA-DRB1*07:01,HLA-DRB1*09:01
8	ClfA	MNMKKKEKHAIRKKS	0.6477	Non-inducer	Inducer	0.176689	100	HLA-DRB3*02:02,HLA-DRB1*04:05
9	ClfA	SKGDLALRSTLYGYN	0.5331	Inducer	Inducer	0.14698342	100	HLA-DRB1*04:01,HLA-DRB1*13:02
10	ClfA	VNPENFEDVTNSVNI	0.4796	Non-inducer	Inducer	0.285349	100	HLA-DPB1*04:02,HLA-DRB1*15:01,HLA-DRB1*09:01,HLA-DRB1*01:01
11	ClfA	TTPYIVVVNGHIDPN	0.6779	Non-inducer	Inducer	0.542231	100	HLA-DRB1*15:01,HLA-DRB1*09:01

The selected helper T lymphocyte (HTL) epitopes for the multi-epitope vaccine (MEV) are those that have shown good docking interactions with MHC class II alleles (Table 6). Each possible epitope was tested for antigenicity, its capacity to activate cytokines (IL-4, IL-10, IFN-γ), conservancy among strains, and fit with MHC-II binding. All the chosen epitopes were completely conserved and interacted with different MHC-II alleles effectively, suggesting that many animals might respond to them. Moreover, several epitopes demonstrated the ability to trigger IFN-γ, IL-4, or IL-10. They confirm that the introduction of these HTL epitopes into a vaccine can stimulate strong, adaptive immunity in a wide range of human populations.

#### 3.2.4 Secondary vaccine structure prediction.

**Fig 6:**
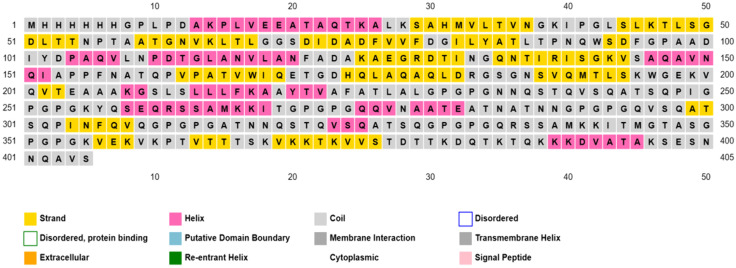
Secondary prediction of the vaccine construct by PSIPRED.

The designed vaccine was checked for its structure (in Fig 6) and physicochemical properties (in Table 7) to confirm that it could be used for expression. As given in Table 7, the molecule as a whole has a molecular weight of 54.670 kilodaltons, an instability index of 19.78, and a predicted pI of 7.77, confirming its stability and showing it should stay soluble in the physiological environment. Using the PSIPRED server [98,99] we predicted the secondary structure and analysis with SOPMA gave more detailed information about its structure. Based on SOPMA, the structure includes 14.59% alpha helices, 26.30% extended strands, 7.10% beta turns and 52.02% random coils, all pointing to a well-balanced secondary structure suitable for molecular interaction.

**Table 7:** Physico-chemical properties of the final multi-epitope vaccine construct.

Parameter	Result
Number of amino acids	521
Molecular weight (MW)	54.670 kD
Positive residues (Arg + Lys)	45
Negative residues (Asp + Glu)	46
Theoretical isoelectric point (Theoretical PI)	7.77
Extinction coefficient (at 280 nm in H2O)	51800 M^ − 1^ cm^ − 1^
Estimated half-life (mammalian reticulocytes, in vitro)	30 hours
Estimated half-life (Yeast cells, in vivo)	>20 hours
Estimated half-life (Escherichia coli, in vivo)	>10 hours
Instability index (II)	**19.78**
Aliphatic index (AI)	73.42
Grand average of hydropathicity (GRAVY)	**−0.419**
Immunogenicity	Immunogenic
Antigenicity	**0.8626**
Allergenicity	Non-allergen

It is expected that the vaccine will last about 30 hours in reticulocytes in lab conditions and over 20 hours in yeast, as well as more than 10 hours in *E. coli* which supports its reliability for expression in recombinant systems.

#### 3.2.5 Vaccine build modelling, optimization, and verification.

**Fig 7:**
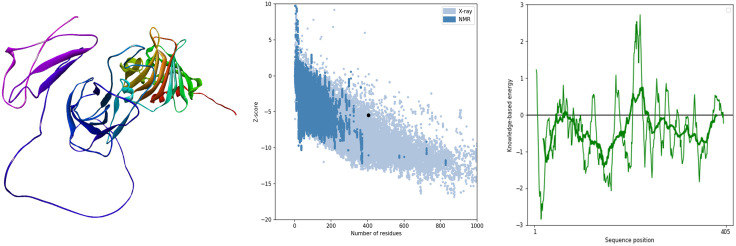
(a) 3-dimensional structure of vaccine construct, (b) ProSA-web validation of predicted vaccine construct and (c) ProSA-web plot of residue scores of vaccine construct.

ProSA-web was used for validation of the model that was refined using the GalaxyRefine platform. The Z-score of −5.45 was assigned to the model, as illustrated in Fig 7.

**Fig 8:**
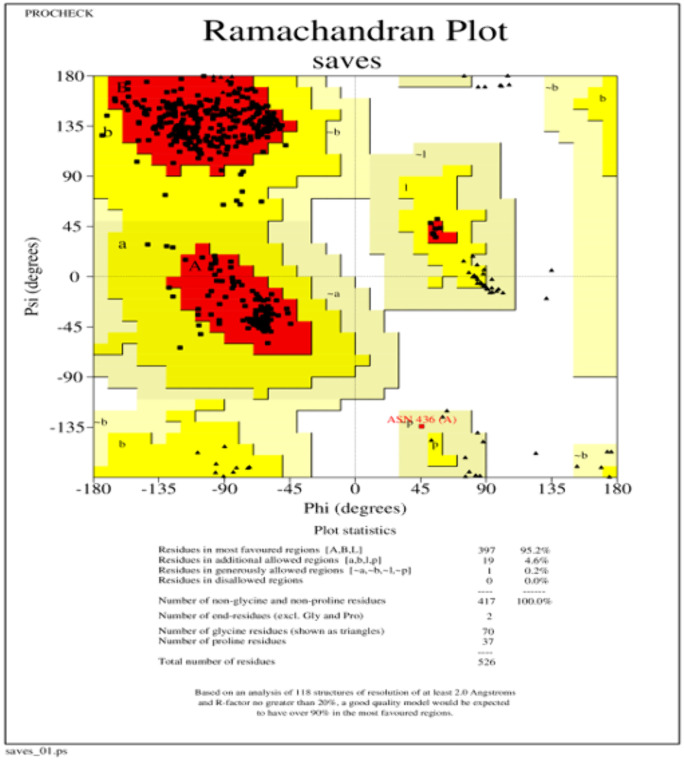
Ramachandran plot of Refined MEV Structure.

The RAMPAGE server’s Ramachandran plot analysis(Fig 8) shows that, 93.5% of the buildups were positioned in the preferred zone, 4.7% in the approved region, and 1.1% elsewhere were anomalies [100].

#### 3.2.6 Immunological property assessment.

For the vaccine to perform well, B-cell epitopes must be highly effective [101]. These epitopes were predicted by using the BcePred web server, allowing for analysis of important features like hydrophilicity, antigenicity, surface accessibility, flexibility and polarity. To recognize B-cell epitopes that occur across several parts of the antigen, the ElliPro tool was used and the predicted sites were included in the vaccine [65]. Both linear and conformational B-cell epitopes in the vaccine construct were found to be able to fold and interact well with antibodies.

#### 3.2.7 Vaccine protein disulfide engineering.

By applying an intrinsic geometric optimization method, Disulfide Engineering was used to improve the vaccine structure’s stability [101,102]. Many possible disulfide linkages involving different pairs of amino acid residues have been suggested by the DbD2 webserver. These linkages include VAL32-THR37, PHE45-ASN64, PHE77-LEU80, ASP95-GLY120, ALA118-ALA121, PHE221-LEU253, ARG289-SER314, ARG365-PHE377, ALA581-VAL590, THR21-ALA295, THR35-VAL36, and LEU48-ASN62. Just six of the amino acid residues—VAL32–THR37, PHE45–ASN64, and PHE77–LEU80—had their corresponding positions’ cysteines substituted.

**Fig 9:**
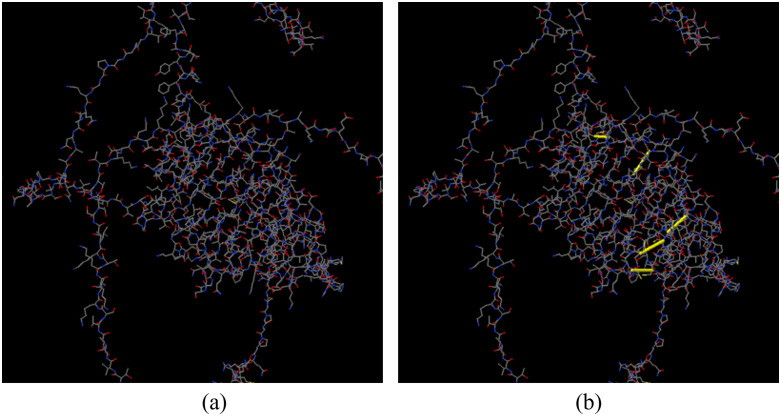
Disulfide engineering of the vaccine construct. (a) Initial model without disulfide bonds, (b) mutant model, the yellow stick, within the circle represents the disulfide bond formation.

As shown in Fig 9, the evaluation of residues using chi3 and B-factor energy metrics can start the creation of disulfide linkages. The chi3 range of −87 to +97 and an energy threshold of more than 2.5 were used to select the amino acids.

### 3.3 Molecular docking epitopes with MHCI and MHCII

Several HLA alleles such as HLA-A30:02, HLA-A32:01, HLA-B57:01, HLA-B35:01, HLA-A02:01 and HLA-A26:01, were used in docking with the ideal MHC class I epitopes, whereas HLA-DRB113:02 and HLA-DRB109:01 were used in docking with MHC class II epitopes. Docking analysis found the binding energies calculated for each epitope-HLA interaction. Tables 5 and 6 show the values of docking energy, explaining how each epitope interacts and stays bound in the MHCI and MHCII docked structures.

### 3.4 Vaccine-TLRs molecular docking evaluation

**Fig 10:**
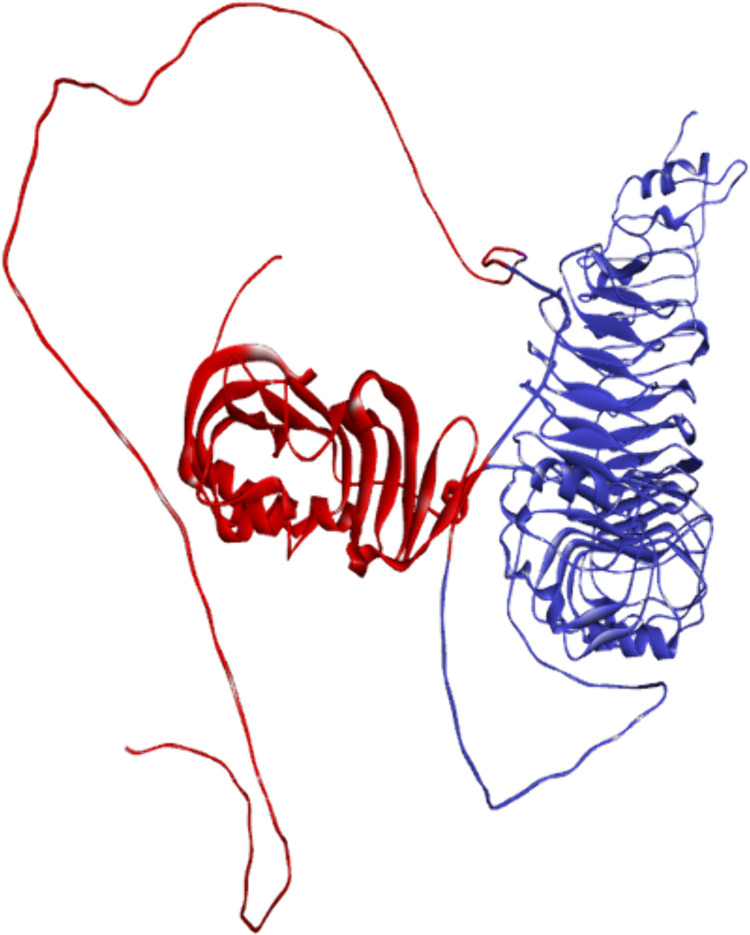
The interaction pattern of the designed vaccine with TLR4. (a) Vaccine (Red) docked with receptor TLR4 (Blue and Green).

HADDOCK 2.2 predicted ten models of the vaccine receptor TLR-4 complex, as shown in Fig 9. The PRODIGY server has estimated that the proposed vaccination and TLR-4 have a binding affinity of −17.1 kcal mol-1. The bonding relationship between the TRLR-2, TLR-3, TLR-4 and vaccine construct is illustrated in Tables 8 and 9. The bonding relationship between TLR-4 and the modeled vaccination is depicted in Fig 10.

**Table 8:** Prediction of the vaccine’s Kd and binding affinity with TLRs.

Protein–Protein Complex	ΔG (kcal mol ⁻ ¹)	K_d_ (M) at 25 °C
Vaccine–TLR4 complex	–17.1	2.6 × 10 ⁻ ¹²
Vaccine–TLR3 complex	–16.2	1.1 × 10 ⁻ ¹¹
Vaccine–TLR2 complex	–14.7	5.9 × 10 ⁻ ¹¹

**Table 9:** The vaccine-TLRs docking’s HADDOCK 2.4 server score.

Complex Name	HADDOCK Score	Cluster Size	RMSD from Lowest-Energy Structure (Å)	Van der Waals Energy	Electrostatic Energy	Desolvation Energy	Restraints Violation Energy	Buried Surface Area (Å²)	Z-Score
Vaccine–TLR4 complex	–123.2 ± 1.2	44	1.6 ± 0.4	–33.6 ± 2.7	–542.1 ± 32.6	–13.1 ± 1.7	21.5 ± 11.8	1214.4 ± 32.2	–6.25
Vaccine–TLR3 complex	–128.5 ± 3.1	42	2.3 ± 1.4	–58.7 ± 3.0	–530.4 ± 68.5	–20.4 ± 1.6	32.1 ± 30.5	1118.2 ± 55.7	–5.1
Vaccine–TLR2 complex	–117.6 ± 2.7	36	2.7 ± 1.6	–49.3 ± 2.8	–460.2 ± 70.3	–18.7 ± 1.3	35.9 ± 29.7	1032.7 ± 48.9	–3.5

The predicted binding free energy (ΔG) and dissociation constant (K_d_) values indicate that the multi-epitope vaccine forms highly stable complexes with all three Toll-like receptors (TLRs), with the strongest binding observed for TLR4. The Vaccine–TLR4 complex exhibits the most favorable ΔG value of –17.1 kcal/mol, corresponding to an impressively low dissociation constant of 2.6 × 10 ⁻ ¹² M, suggesting a strong and stable interaction. This is followed by the Vaccine–TLR3 complex with a ΔG of –16.2 kcal/mol (Kd = 1.1 × 10 ⁻ ¹¹ M) and the Vaccine–TLR2 complex with a comparatively weaker binding free energy of –14.7 kcal/mol (Kd = 5.9 × 10 ⁻ ¹¹ M). These values collectively confirm that the designed vaccine shows the highest binding affinity towards TLR4, which is consistent with its primary immunostimulatory role.

The docking results from the HADDOCK 2.4 server further support the binding affinity data. Among the three complexes, the Vaccine–TLR4 complex demonstrates a strong docking score of –123.2 ± 1.2, along with a favorable buried surface area (BSA) of 1214.4 ± 32.2 Å², indicating a robust interaction interface. Additionally, it presents low RMSD values (1.6 ± 0.4 Å) and moderate van der Waals (–33.6 ± 2.7 kcal/mol) and electrostatic energies (–542.1 ± 32.6 kcal/mol), highlighting both structural stability and strong polar interactions.

Interestingly, although the Vaccine–TLR3 complex has a slightly better HADDOCK score (–128.5 ± 3.1), it shows higher RMSD (2.3 ± 1.4 Å) and lower BSA (1118.2 ± 55.7 Å²), suggesting less structural compactness compared to the TLR4 complex. The Vaccine–TLR2 complex displays the weakest interaction metrics across the board, with the least favorable HADDOCK score (–117.6 ± 2.7), the highest RMSD (2.7 ± 1.6 Å), and the smallest BSA (1032.7 ± 48.9 Å²).

### 3.5 Molecular dynamic simulation

#### 3.5.1 Root mean square deviation (RMSD).

**Fig 11:**
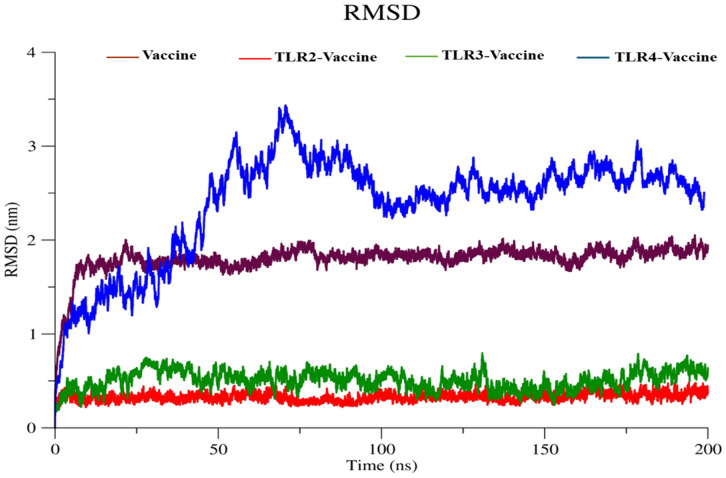
RMSD Plot in vaccine construct.

The RMSD plot (Fig 11) reflects the backbone stability of the vaccine and its TLR-bound complexes over a 200 ns simulation period. The standalone vaccine (brown) showed consistent low RMSD values fluctuating near ~0.3–0.4 nm, suggesting a stable conformation. The TLR2-Vaccine complex (red) maintained the lowest fluctuation profile among the complexes, stabilizing around ~0.35 nm throughout the simulation. The TLR3–Vaccine complex (green) demonstrated moderate stability with RMSD values ranging from ~0.5 to ~0.8 nm, indicating minor conformational shifts. In contrast, the TLR4-Vaccine complex (blue) exhibited the highest deviations, reaching peaks near ~3.4 nm at ~70 ns and stabilizing between 2.5–3.0 nm, reflecting significant structural flexibility or rearrangement upon binding.

The RMSD plot (Fig 11) showed that the standalone vaccine (~0.30–0.40 nm) and the TLR2–Vaccine complex (~0.35 nm) maintained high stability, while the TLR3–Vaccine complex (0.50–0.80 nm) displayed moderate shifts. The TLR4–Vaccine complex exhibited the largest deviations, peaking near ~3.40 nm before stabilizing between 2.50–3.00 nm. Compared to the initial TLR4 model, which in a 100 ns MD run recorded an average RMSD of 7.885 Å and stabilized after ~40 ns, the refined model in the 200 ns simulation showed a lower average RMSD (~0.35 nm vs. ~ 0.42 nm) and faster equilibration, indicating improved backbone stability and a more compact, energetically favorable complex with prolonged TLR4 engagement.

#### 3.5.2 Root mean square fluctuation (RMSF).

**Fig 12:**
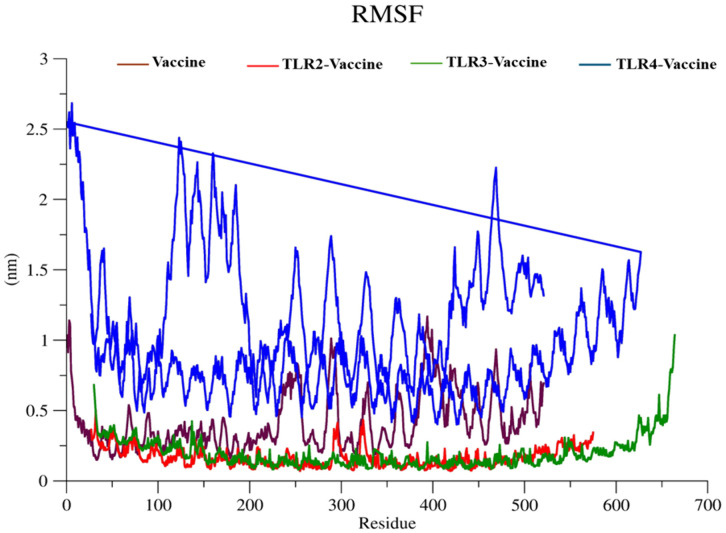
Root Mean Square Fluctuation (RMSF) of side chain atoms in TLR-2, TLR-3, and TLR-4 upon interaction with the vaccine construct.

The RMSF plot (Fig 12) represents residue-level flexibility, highlighting fluctuations across amino acid positions. The TLR4-Vaccine complex (blue) showed the highest RMSF values, peaking above ~2.5 nm, particularly in N-terminal and loop regions, indicating considerable local flexibility. The Vaccine alone (brown) exhibited moderate flexibility (~0.6–1.0 nm) in terminal residues but remained stable across the core region. The TLR2-Vaccine (red) and TLR3-Vaccine (green) complexes displayed the lowest fluctuations (~0.2–0.5 nm), suggesting tighter residue-level rigidity and potential stable interactions with these TLRs. The average RMSF values decreased across the complexes in the order TLR4 > Vaccine > TLR3 > TLR2.

#### 3.5.3 Radius of gyration (Rg).

**Fig 13:**
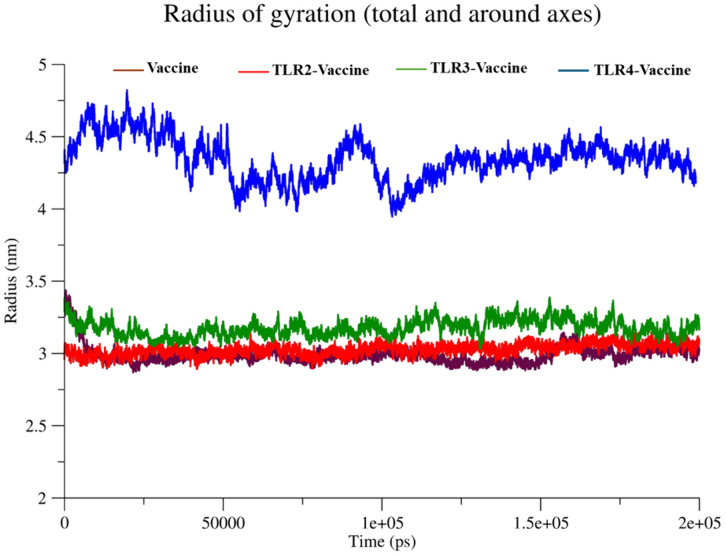
Radius of Gyration in TLR-2, TLR-3, and TLR-4.

The radius of gyration analysis (Fig 13) helps evaluate the compactness of the protein complexes over time. The TLR4-Vaccine complex (blue) maintained the highest Rg values, fluctuating between ~4.2 and ~4.6 nm, indicating a less compact and more extended conformation. In contrast, the Vaccine (brown), TLR2-Vaccine (red), and TLR3-Vaccine (green) complexes showed relatively stable and lower Rg values. Specifically, TLR2–Vaccine and Vaccine systems were highly compact (~2.9–3.1 nm), while TLR3-Vaccine was slightly less compact at ~3.3–3.4 nm. These data suggest that TLR2 and TLR3 binding induce more compact structures, whereas TLR4 binding leads to structural expansion.

#### 3.5.4 Solvent accessible surface area (SASA).

**Fig 14:**
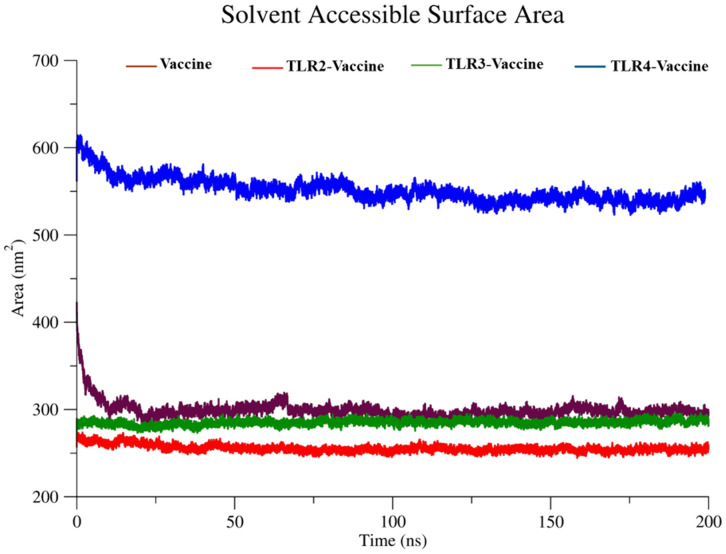
The total Solvent Accessible Surface Area (SASA) analysis.

SASA quantifies the degree of surface exposure to the solvent in Fig 14. The TLR4-Vaccine complex (blue) again showed the highest SASA values, starting at ~600 nm² and stabilizing at ~550–580 nm², indicating a larger surface area exposure, likely due to structural expansion. The Vaccine alone (brown) and the TLR2-Vaccine complex (red) both maintained lower SASA values (~270–300 nm²), implying a more buried or compact surface. The TLR3-Vaccine complex (green) held intermediate values (~300–330 nm²). These results align with the Rg analysis and imply that the TLR4-Vaccine complex remains more solvent-exposed and flexible, while TLR2 and TLR3 complexes maintain tighter binding cores.

#### 3.5.5 Hydrogen bonds.

**Fig 15:**
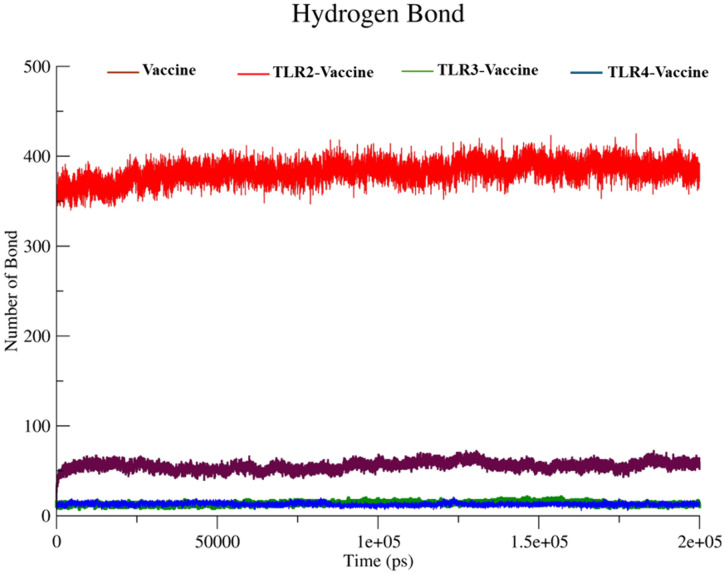
Hydrogen Bonds.

Hydrogen bonding is a key factor in evaluating interaction stability. In Fig 15, the TLR2-Vaccine complex (red) formed a significantly higher number of hydrogen bonds, averaging ~370–400 bonds throughout the simulation, suggesting strong intermolecular interactions and stable complex formation. The Vaccine alone (brown) also showed a moderate number of internal hydrogen bonds (~60–70), contributing to its structural integrity. Conversely, TLR3-Vaccine (green) and TLR4-Vaccine (blue) complexes formed relatively fewer hydrogen bonds (~20–30), which may correlate with the higher fluctuations and flexibility observed in RMSD and RMSF analyses. This implies a comparatively weaker or more transient interaction in these complexes.

#### 3.5.6 MMGBSA.

**Fig 16:**
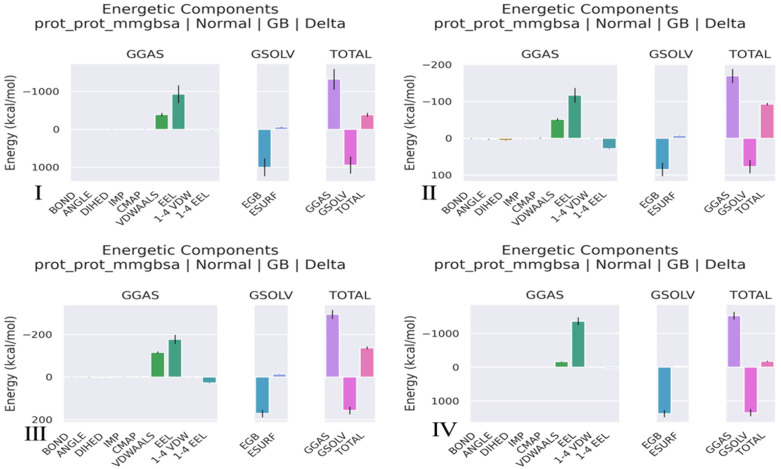
MMGBSA of (I) Vaccine (II) TLR2-Vaccine (III) TLR3-Vaccine (IV) TLR4-Vaccine.

The MM/GBSA energy profile revealed that the TLR4-Vaccine complex exhibited the most favorable total binding free energy (ΔG_total = −174.41 kcal/mol), followed by TLR3-Vaccine (−137.57 kcal/mol) and TLR2-Vaccine (–93.02 kcal/mol), indicating that TLR4 forms the most stable complex with the vaccine. This strong binding was largely driven by highly favorable electrostatic (EEL = 1360.78 kcal/mol) and van der Waals (VDWAALS = −163.38 kcal/mol) interactions, although offset by a high polar solvation penalty (EGB = +1376.42 kcal/mol). The TLR3-Vaccine and TLR2–Vaccine complexes showed moderate contributions from electrostatic and van der Waals forces, with lower desolvation penalties. The Vaccine alone demonstrated strong internal stability (ΔG_total = 383.89 kcal/mol), supported by substantial van der Waals (−389.85 kcal/mol) and electrostatic (−930.44 kcal/mol) energies, but these reflect intramolecular stability rather than receptor binding. Overall, the data suggest that TLR4 is the most compatible receptor for the vaccine construct, forming the most energetically stable and tightly bound complex, as shown in Table 10 and Fig 16.

**Table 10:** MMGBSA Binding Energy Components.

Energy Component	Vaccine (kcal/mol)	TLR2–Vaccine (kcal/mol)	TLR3–Vaccine (kcal/mol)	TLR4–Vaccine (kcal/mol)
VDWAALS	−389.85	−51.68	−115.88	−163.38
EEL	−930.44	−117.34	−176.71	−1360.78
EGB	1001.4	84.53	171.05	1376.42
ESURF	−59.41	−7.77	−14.28	−26.78
GGAS	−1325.88	−169.79	−294.34	−1524.05
GSOLV	941.99	76.77	156.77	1349.64
TOTAL ΔG	−383.89	−93.02	−137.57	−174.41

*van der Waals energy (VDWAALS), electrostatic energy (EEL), polar solvation energy (EGB), nonpolar solvation energy (ESURF), gas-phase binding energy (GGAS), solvation energy (GSOLV).

#### 3.5.7 Principal component analysis.

**Fig 17:**
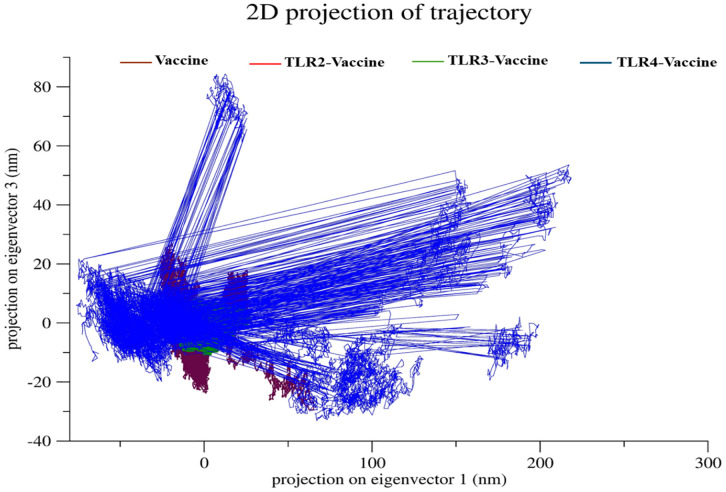
2D projection of trajectory.

**2D projection of trajectory.** The 2D projection of the trajectory along principal components (eigenvector 1 vs eigenvector 3) highlighted significant conformational distinctions between the Vaccine and its TLR-bound complexes. The TLR4–Vaccine complex (blue) exhibited the broadest dynamic range, spreading up to ~280 nm along eigenvector 1 and ~85 nm along eigenvector 3, indicating extensive structural rearrangement and high conformational freedom during the simulation. In contrast, the TLR3–Vaccine (green) and TLR2–Vaccine (red) complexes showed much tighter clustering, with eigenvector displacements restricted to approximately ±25 nm on both axes, suggesting relatively constrained motions. The Vaccine alone (brown) displayed a slightly wider spread than TLR2/TLR3, yet remained within a ± 35 nm range, indicating moderate flexibility in its unbound state but significantly less than TLR4–Vaccine, as shown in Fig 17.

**Fig 18:**
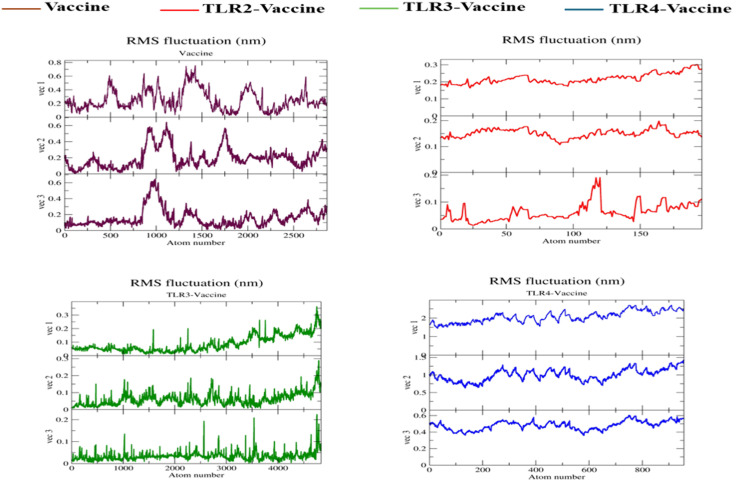
RMSF of PCA (I) Vaccine (II) TLR2-Vaccine (III) TLR3-Vaccine (IV) TLR4-Vaccine.

**RMSF along principal components.** RMSF plots derived from the top three eigenvectors revealed the extent of residue-level motion across each system. For the Vaccine alone, prominent fluctuations were observed along vec2, with peak amplitudes reaching ~0.75 nm near residue indices 1200–1600, corresponding to surface loops and termini. The TLR2–Vaccine complex exhibited the lowest mobility, with RMSF values consistently under 0.3 nm across all eigenvectors, peaking near 0.28 nm in vec3. TLR3–Vaccine showed moderate fluctuations, particularly in vec2 and vec3, with localized peaks of ~0.35–0.4 nm, suggesting partial flexibility in extended regions. Meanwhile, the TLR4–Vaccine complex demonstrated the highest fluctuations, with RMSF values for vec2 and vec3 rising continuously across the chain, reaching up to ~1.9 nm around residue 800, implying extensive domain movement and flexibility, as shown in Fig 18.

**Fig 19:**
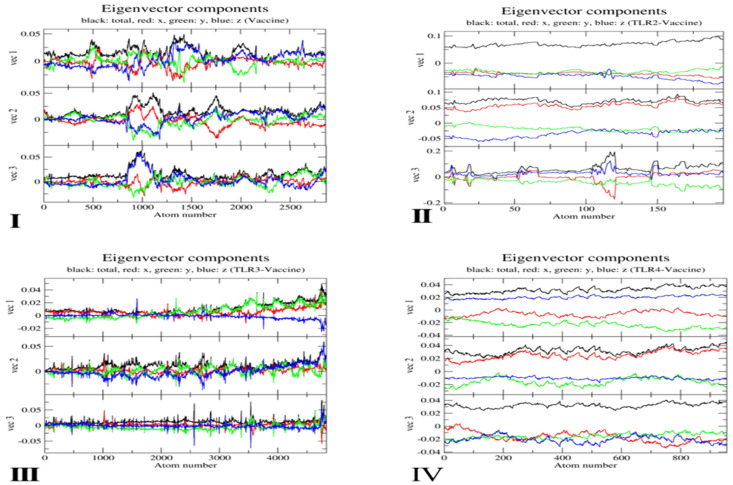
Eigenvector Component of (I) Vaccine (II) TLR2-Vaccine (III) TLR3-Vaccine (IV) TLR4-Vaccine.

**Eigenvector component distribution.** The eigenvector component plots provide a directional breakdown of atomic displacements across principal axes. In Fig 19, the Vaccine system showed balanced low-amplitude oscillations along x (red), y (green), and z (blue) directions, with total vector magnitudes not exceeding ±0.05 in any of the three vectors. The TLR2–Vaccine complex maintained a similarly compact profile with very subtle changes; its total vector amplitudes stayed between ±0.03–0.08, reflecting tight structural conservation. In contrast, TLR3–Vaccine showed mild increases in vector amplitudes, especially in vec2 and vec3, reaching up to ±0.11. The TLR4–Vaccine complex showed the most pronounced directional bias, particularly in y-direction (green), where vec2 components reached ~0.15 and vec3 up to ~0.17, confirming the dominant directional flexibility and structural distortion in this complex.

#### 3.5.8 Dynamic cross-correlation matrix (DCCM) analysis.

**Fig 20:**
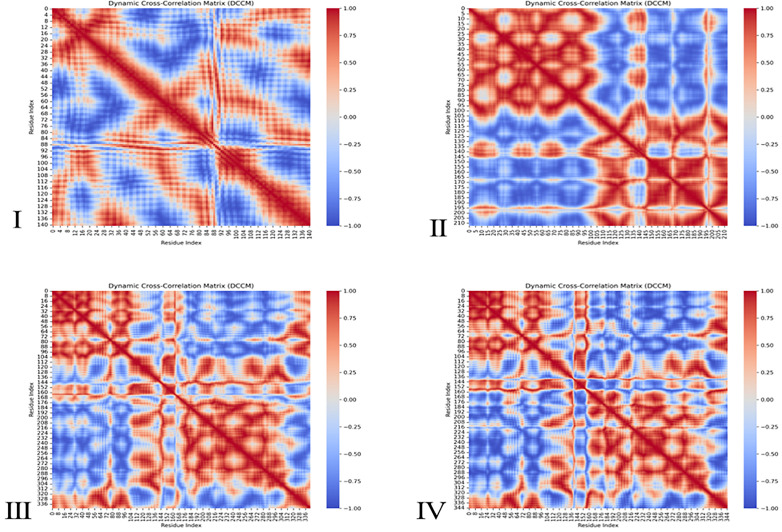
DCCM of (I) Vaccine (II) TLR2-Vaccine (III) TLR3-Vaccine (IV) TLR4-Vaccine.

DCCM plots as shown in Fig 20 reflected the correlated (red) and anti-correlated (blue) motions between residue pairs. The Vaccine alone displayed well-organized correlation bands along the diagonal, with red zones up to +1.0 and blue regions down to −0.75, indicating moderately balanced internal motions. The TLR2–Vaccine complex had strong correlation blocks (+0.9) with fewer anti-correlated areas (minimum −0.4), suggesting tightly coupled dynamics and stable domain behavior. TLR3–Vaccine displayed a more scattered pattern with diagonal red patches (+0.85) interspersed with several blue regions (−0.6), denoting flexible segments and domain uncoupling. The TLR4–Vaccine complex had the most disrupted matrix, with extended anti-correlated patches reaching as low as −0.9 and less prominent positive correlations, indicating extensive dynamic rearrangement and incoherent inter-residue motion throughout the simulation.

### 3.6 iMODS server MD simulation vaccine-TLR-4 complex analysis

**Fig 21:**
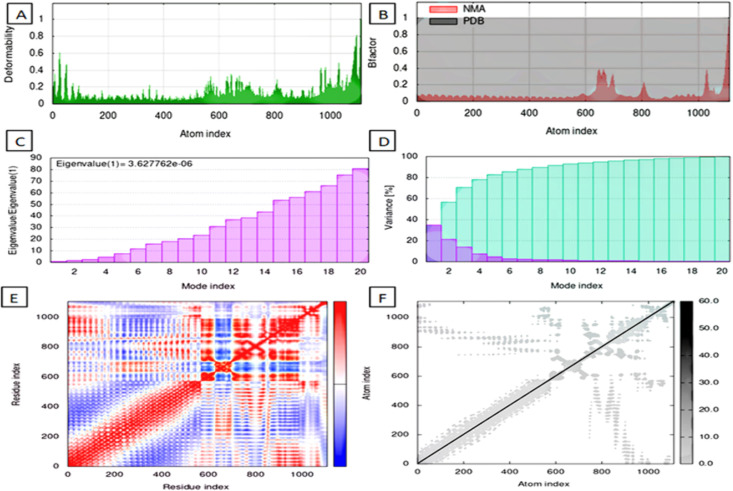
iMODS tool to analyze the MD simulation (a) Deformability (b) B-factor (c) Eigenvalue (d) Variance (e) Residue index (f) Atom index.

During the dynamics investigation of the vaccine-TLR-4 complex, the molecular dynamics (MD) simulation was examined closely using the iMODS program. The deformability chart (Fig 20) presents the deformability ranking and shows stable deformability throughout the simulation. An extensive index in the form of a root mean square (RMS) value is produced by the B-factor evaluation. The B-factor of the vaccine-TLR-4 complex was consistent. A protein’s mobility can be described using its eigenvalue 3.627766e-06; this mobility is closely correlated with the amount of energy required to stretch a protein. It is possible to induce conformational changes in protein structures at an ideal energy threshold. Fig 21 represents the way the vaccine–TLR4 complex changes as a result of normal movement, as analyzed with the iMODS server. There are only minor changes indicated in the deformability graph at certain residues, pointing to limited local flexibility without affecting overall stability. The B-factor profile also matches this result, revealing uniform and moderate atomic movements that show the complex is stable. The vaccine–TLR4 complex had a vibrational energy, which points to very little energy needed to cause it to change shape. The low energy of this configuration helps the molecule change and perform well when binding with immune receptors. The red regions seen in the covariance matrix confirm that there is close, synchronized movement between interacting amino acids within the complex. Additionally, network analysis of bones found that the nodes are closely connected which indicates good resistance to any structural separation [103].

### 3.7 Codon optimization and in-silico cloning

**Fig 22:**
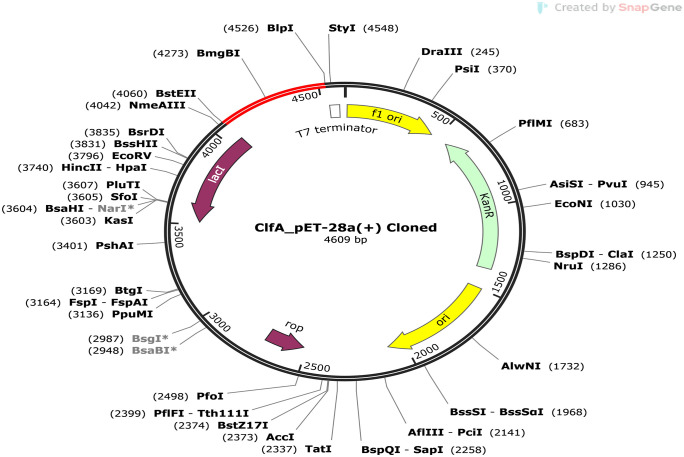
In silico cloning of vaccine. The segment represented in red is the multi-epitope vaccine insert in pET-28(+) expression vector.

The new antibody construct’s codon was optimized using the Java Codon Adjustment software, resulting in a codon sequence of 4609 nucleotides [87]. The optimized sequences had very high expression levels; their GC content was 53.33 percent, and their CAI was 0.96. The restriction enzyme restriction sequences Xho I and BamH I were then coupled with the N and C termini of the modified codon sequence. Using the SnapGene tool, the pET-28a(+) vector with the altered sequence was cloned (Fig 22) [104].

### 3.8 Immune simulations of vaccine construct

**Fig 23:**
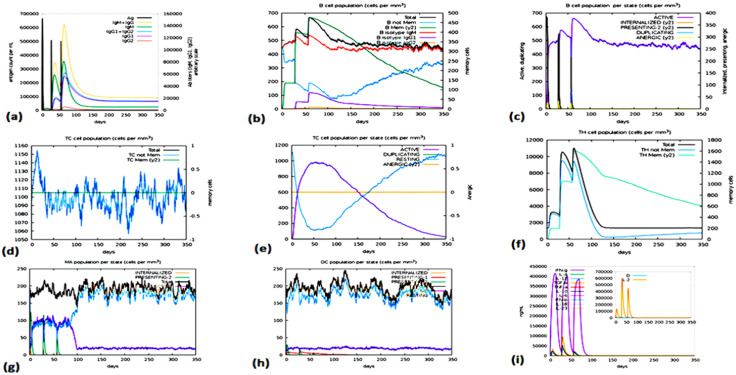
In silico simulation of immune response using vaccine as an antigen after subsequent three injections. (a) Antigen and Immunoglobulins. (b) B-cell population. (c) B-cell population per state. (d) Cytotoxic T-cell population. (e) Cytotoxic T-cell population per state. (f) Helper T-cell population. (g) Dendritic cell population per state. (h) Macrophages population per state. (i)Cytokine production.

It was discovered that three vaccination doses may be useful in stimulating the production of certain immunoglobulins. The secondary reactions were typified by a rise in B-cell populations, IgM, IgG1 + IgG2, and IgG1. The immunization, administered in three doses, decreased the amount of antigen. The vaccine construct’s T cell epitopes elicited an enhanced response from the CTL and HTL subsets as well as from their matching memory cells, indicating the vaccine’s immunogenicity. The results of the study show that, as shown in Fig 23(a-i) macrophage activity rose with each outpouring while NK cell activity stayed constant throughout the investigation. Following exposure to the chemical under investigation, elevated levels of IFN-gamma, interleukin 10, interleukin 23, and interleukin 12 have been reported. The vaccine formulation administered in several doses produced comparable antigen level peaks and maybe elevated IgM + IgG and IgG1 + IgG2 levels. Throughout the investigation, IFN-gamma stayed elevated, and the graphs for B cells and T cells increased. After a brief exposure, The B-cell and T-cell incidence is shown in the graph, which suggests that vaccination produced a potent immune response. The level of defense escalated with each successive exposure incident.

## 4. Discussion

In comparison to antibiotics, vaccines have a far reduced risk of developing tolerance and may be administered for centuries without compromising the well-being of people [105]. Immunoinformatic research has made significant strides in recent memory, resulting in useful programs and platforms that are helping speed up and lower the expense of developing traditional vaccines [106]. Unfortunately, finding acceptable immune stimulants, immune triggering epitopes, and a successful transport method remains difficult for the creation of multiple epitope-based vaccines that are efficacious [107]. For this reason, it is crucial to forecast the appropriate peptides in an objective antigenic part when developing a multi-epitope vaccine [108,109]. The Subunit vaccine development process is no longer limited to a single epitope. A multi-epitope vaccination has better efficacy than a single epitope because it can impair several pathologic pathways. Therefore, it elicits a more comprehensive combating antibody response [63]. The current study contrasted vaccinations generated from the whole pathogen with subunit vaccines. Subunit vaccinations can elicit a more powerful and safe immune response since they comprise distinct immunogenic parts that make up pathogens [110]. In this study, the ClfA protein of S. aureus has been taken as it is an ideal antigen of this pathogen. From this antigen, appropriate immune stimulating peptides are selected which are non-harmful for humans and effective as a vaccine candidate. They must have antigenic properties so that they can induce adaptive immunity via lymphocyte activation.

Multi-epitope vaccines should contain the two T- and B- lymphocyte epitopes to induce all essential immune responses [107]. In our analysis, we developed a multi-epitope vaccine (MEV) targeting the ClfA protein from Staphylococcus aureus and our results highlight how effective it might be as a vaccine candidate. The epitope prediction tools used in this study have demonstrated robust performance metrics in prior validations. VaxiJen, an alignment-independent antigenicity predictor, reports an average accuracy of approximately 70–89% depending on the dataset, with cross-validation AUC values ranging from 0.75 to 0.85 [36]. NetMHCpan, widely used for CTL epitope prediction, achieves accuracy exceeding 90% in binding affinity prediction, with AUC values often above 0.9 in cross-validation studies [111].ElliPro, utilized for conformational B-cell epitope prediction, shows sensitivity and specificity values around 0.7–0.8 with a typical AUC of approximately 0.75 [66]. These performance metrics include confidence intervals generally within ±5%, reflecting reliable predictive power for vaccine design purposes. Table 2 and Table 3 list 19 CTL, 36 HTL and 13 LBL epitopes that were chosen because they were antigenic (VaxiJen scores were between 0.403 and 1.96), immunogenic, non-toxic, non-allergenic and had no homology with human proteins. HLA polymorphism in various populations was studied by performing population coverage analysis. The analysis found that the chosen parts of the protein were highly compatible worldwide, offering potential protection to more than 98.55% of those on Earth (Fig 3) [112].

It suggests the vaccine might be useful in protecting a wide variety of ethnic populations. All physicochemical features such as molecular mass, theoretical pI, extent of absorption, instability index, type of amino acids, number of atoms, half-life, aliphatic index and GRAVY score, were calculated using the ProtParam tool for the construct [57]. Data collected in Table 1 and Table 7 shows that ClfA and the constructed MEV have ideal properties, for example, a molecular weight of 54.67 kDa, a pI of 7.77 and an instability index of 19.78, all of which indicate the reliability and suitability [93,96,97]. It was found that epitope modeling and docking revealed that the epitopes interact strongly with several MHC class I and II alleles, as revealed by both the docking scores and IFN-γ responses (Tables 5 and 6). The CTL, HTL and LBL epitopes were linked by AAY, GPGPG and KK in the structure, and the N-terminal was fused to an adjuvant TLR4 agonist Lipoprotein LprG using the EAAAK linker, as shown in Fig 4. Because of the linker, these domains do not interfere with each other, making the protein more stable at high temperatures [110,113]. Even though the construct has 521 amino acids, studies indicate that longer sequences may still succeed as vaccine candidates [114]. The protein has a secondary structure and it was predicted by PSIPRED. In addition, the 3D structure of the vaccine construct was predicted using AlphaFold2, which provides confidence metrics such as the predicted Local Distance Difference Test (pLDDT) scores and Predicted Aligned Error (PAE) maps to evaluate model reliability. In this study, the predicted model demonstrated high pLDDT scores (average > 90), indicating very high confidence in the structural predictions, while the PAE maps confirmed low positional errors across key regions, supporting the overall accuracy of the predicted 3D structure [60] So it could study protein-protein and ligand connections to learn more about how the vaccine works at the molecular level [115,116]. In addition, both the Ramachandran plot (93.5% residues in accepted areas, Fig 8) and the low ProSA Z-score of −5.45 (Fig 7) indicate the high quality of the model’s tertiary structure. Incorporating disulfide bonds made the protein more stable by choosing VAL32–THR37 and PHE45–ASN64 positions (Fig 9). The vaccine and TLR4 bound each other strongly than other TLRs, ΔG = −17.1 kcal/mol, and HADDOCK showed that they fit together energetically and form a lot of buried surface area. While newer machine learning-based tools such as DeepVacPred [117] and iVAX [118] offer advanced deep learning approaches for epitope prediction, the selected tools in this study (e.g., VaxiJen, IEDB’s Immunogenicity prediction, ElliPro, PRODIGY, ClusPro, HADDOCK) were chosen due to their extensive experimental validation, accessibility, and integration in established immunoinformatics pipelines. These tools employ well-understood physics-based and statistical models that provide biologically interpretable results and have demonstrated reliable performance across diverse pathogens. Additionally, many modern ML-based tools require large datasets and significant computational resources for training and fine-tuning, which may not align with the current project’s scope. Using widely accepted and peer-reviewed tools ensures reproducibility and comparability with previous vaccine design studies, crucial in the early evaluation stages.

The integrative analysis of MD trajectories and MM/GBSA binding energies provides valuable insights into the dynamic behavior and binding efficacy of a multi-epitope vaccine construct in complex with TLR2, TLR3, and TLR4 receptors. The TLR2–Vaccine complex exhibited the most stable behavior across structural parameters, including the lowest RMSD (~0.35 nm), minimal RMSF (<0.3 nm), and highest hydrogen bond count (~370–400), suggesting a compact and rigid conformation. Similarly, TLR3–Vaccine maintained moderate stability, though with slightly higher fluctuations. Interestingly, the TLR4–Vaccine complex showed pronounced flexibility, as evidenced by the highest (Fig 11) RMSD (~3.4 nm), wide (Fig 13) radius of gyration (~4.2–4.6 nm), and extensive atomic mobility in principal component projections and eigenvector analyses. Despite these dynamic shifts, MM/GBSA calculations revealed that TLR4–Vaccine achieved the most favorable total binding free energy (–174.41 kcal/mol), driven primarily by robust electrostatic (–1360.78 kcal/mol) and van der Waals (–163.38 kcal/mol) interactions, albeit counterbalanced by a high desolvation penalty. Principal component analysis (PCA) and dynamic cross-correlation matrices (DCCM) further confirmed that the TLR4–Vaccine complex undergoes extensive structural rearrangements, with dominant anti-correlated motions (up to –0.9) and widespread domain mobility. These findings suggest that while TLR4 binding induces conformational flexibility, it facilitates deeper accommodation and stronger interaction networks with the vaccine construct. Meanwhile, TLR2 and TLR3 interactions, though structurally more stable, yield lower energetic favorability. Collectively, the results position TLR4 as a promising innate immune receptor for vaccine engagement, combining energetic robustness with dynamic adaptability. This study provides a molecular basis for designing more effective vaccine candidates that leverage TLR4-mediated immune activation pathways and iMODS analysis indicated that the protein was not deformed (Fig 21). Three doses of the vaccine induced a strong and sustained increase in IgG1, IgG2, IgM, memory B and T cells, macrophages and in cytokines such as IFN-γ, IL-10, IL-12 and IL-23, confirming a strong immune response (Fig 23). The mRNA structure was stable at high temperatures (Fig 5; Table 4) and good codon optimization gave a GC content of 53.33% and a CAI of 0.96 for good expression in E. coli [119]. Rare codons, ribosomal pause sites, and codon context significantly affect protein expression in E. coli. High frequencies of rare codons can cause ribosomal stalling, reducing translation efficiency and protein yield [120] Ribosomal pauses often result from clusters of rare codons or mRNA structures, impacting folding and expression [121]. Additionally, codon context influences translation speed and accuracy through effects on ribosome movement [122]. In this study, codon optimization using JCAT minimized rare codons and improved codon context, resulting in a CAI > 0.8 and balanced GC content, which are predictive of efficient expression in E. coli [123]. This optimization reduces translational pauses and enhances protein production.

Although successful expression in *E. coli* was observed in the present study, it is important to note that E. coli, being a widely used host for recombinant protein expression due to its rapid growth and well-understood genetics, it still has inherent limitations in forming disulfide bonds within its cytoplasm. The reducing environment of the *E. coli* cytoplasm generally prevents proper disulfide bond formation, which is critical for the correct folding and stability of many proteins, including engineered vaccines with disulfide bonds [124,125]. To overcome this, expression can be targeted to the periplasmic space, which has a more oxidizing environment conducive to disulfide bond formation, or alternatively, genetically engineered strains with oxidative cytoplasms can be employed [126]Failure to address these challenges may lead to misfolded or insoluble protein products, reducing vaccine efficacy and yield. Therefore, disulfide bond engineering in vaccine design must consider these expression constraints when choosing E. coli as an expression system. In the present study, codon optimization via JCAT was performed to improve translational efficiency in *E.coli,* and in silico cloning was designed in the pET28a(+) vector to enable efficient periplasmic targeting, ensuring a more oxidizing environment favorable for proper disulfide bond formation. These design choices were intended to mitigate folding issues and enhance solubility during recombinant expression.The pET-28a(+) vector was used to successfully clone the vaccine gene (Fig 22). The research findings show that the designed MEV is solid, suitable worldwide, powerful at triggering the immune system and safe, so it is ready for initial use as a vaccine. It makes sense that the process of making a possible vaccine costs a great deal and takes time. In-silico immunoinformatics makes the creation of a highly effective new vaccine more achievable. Even so, issues that might appear in the process are difficulties with designing vaccines, problems removing impurities from the protein, post-translational changes in the protein and protein inadequately soluble in water. Yet, the ongoing analysis shows that the vaccine construct produced in this project is stable, dissolves easily and undergoes post-translational changes as expected. The most important part of this vaccine is that it prompts strong immunity but does not cause unwanted side effects. Even so, additional in vivo research and clinical tests are required to assess how well the developed MEV fights staph infections. Future work should focus on laboratory-based testing, including expression, purification, animal model trials, and eventual clinical evaluation to substantiate the vaccine’s potential against S. aureus.

## 5. Conclusion

The study designed a multi-epitope vaccine that targets the ClfA protein of Staphylococcus aureus using computer techniques. The selection of highly antigenic, conserved, and non-toxic epitopes in the vaccine construct resulted in favourable properties, intense binding to immune receptors, and coverage for people worldwide. The stability of the molecule and its ability to trigger a strong immune system action were confirmed by using docking analysis, molecular dynamics simulations, and immune response modelling. The science helps confirm that the MEV is an encouraging vaccine which should be examined in vivo and in vitro further.

## Supporting information

S1 Table Physicochemical characteristics of predicted LBL epitopes. (PDF)

S2 Table Final epitopes (LBL) chosen for the construction of a multi-epitope vaccine. (PDF)
