# Supplementary material for: A multi-epitope vaccine targeting a key virulence factor ClfA: An In-silico approach to combat Staphylococcus aureus infections
Source: PLoS One. 2025 Oct 24;20(10):e0334885. doi: 10.1371/journal.pone.0334885 (PMC12551870; doi:10.1371/journal.pone.0334885)
Supplement: S1 Table — (PDF) [file pone.0334885.s001.pdf]

S1 Table: Physicochemical characteristics of predicted LBL epitopes.

Epitope	Antigenicity	Allergenicity	Toxicity	Homology	Immunogenicity
DQTEKVPGKPGIKNPD	0.6143	No	Non-Toxin	Non-Homologue	0.52846193
EKTITTPTLKNPLTGE	0.5054	No	Non-Toxin	Non-Homologue	0.074946237
EKTITTPTLKNPLTGV	0.5054	No	Non-Toxin	Non-Homologue	0.50804562
HFEGYGSVDIQKKPTD	0.5878	No	Non-Toxin	Non-Homologue	0.1237901
EKVGEGQPTEEITKQP	1.1444	No	Non-Toxin	Non-Homologue	0.5952853
GEKEEVPGKPGIKNPE	1.0242	No	Non-Toxin	Non-Homologue	0.65322953
