# Supplementary material for: A multi-epitope vaccine targeting a key virulence factor ClfA: An In-silico approach to combat Staphylococcus aureus infections
Source: PLoS One. 2025 Oct 24;20(10):e0334885. doi: 10.1371/journal.pone.0334885 (PMC12551870; doi:10.1371/journal.pone.0334885)
Supplement: S2 Table — (PDF) [file pone.0334885.s002.pdf]

S2 Table: Final epitopes (LBL) chosen for the construction of a multi-epitope vaccine.

Epitope	Antigenicity	Allergenicity	Toxicity	Homology	Immunogenicity
TLTMPAYIDPENVKKTK	0.54432	Non-Allergen	Non-Toxic	Non-Homologue	0.0787623
DDVKATLTMPAYIDP	0.32234	Non-Allergen	Non-Toxic	Non-Homologue	0.873324
